# Design and Experimental Investigation of a Multi-Level Heartbeat Sound Feedback-Based Neurofeedback System: Neural Mechanisms

**DOI:** 10.3390/s26103187

**Published:** 2026-05-18

**Authors:** Xiuyan Hu, Mingge Kang, Yijing Liu, Ting Shi, Xinyu Shi, Yunfa Fu, Anmin Gong

**Affiliations:** 1 College of Information Engineering, Chinese People’s Armed Police Force Engineering University, Xi’an 710086, China; huxiuyan2024@163.com (X.H.); 15029751650@163.com (M.K.); 13978007608@139.com (Y.L.); lovestforever1209@163.com (T.S.); 18729090681@163.com (X.S.); 2 School of Information Engineering and Automation, Kunming University of Science and Technology, Kunming 650500, China; fyf@ynu.edu.cn; 3 School of Life Science and Technology, Xi’an Jiaotong University, Xi’an 710049, China

**Keywords:** neurofeedback training, brain–computer interface, heartbeat sound feedback, neural mechanisms, dual-task interference, frontal midline theta, cross-frequency coupling, functional connectivity, individual differences

## Abstract

Auditory neurofeedback training (NFT) based on brain–computer interfaces (BCIs) has recently entered the precision motor domain as a task-embedded neural state regulation paradigm. Compared to traditional standalone NFT approaches (e.g., relaxation or attention training designed to enhance general cognitive abilities), task-embedded paradigms integrate feedback directly into the motor task execution process. However, this design inevitably creates a dual-task scenario, and the effects of such a scenario on neural activity and behavioral performance have received limited systematic investigation in the existing literature. This study designed and implemented a closed-loop BCI system employing five-level heartbeat sound feedback and used this system as a research platform to examine the immediate neural mechanism changes and potential dual-task interference effects induced by single-session auditory NFT in moderately skilled shooters. The system maps real-time EEG features onto graded auditory signals varying in playback rate and volume intensity, incorporating a dynamic threshold adjustment mechanism. Twenty-two moderately skilled shooters completed three within-subject conditions (no-sound baseline, SMR enhancement, and theta suppression) in a single session with 32-channel EEG and behavioral data recorded simultaneously. Analyses employed whole-brain cluster-based permutation tests, cross-frequency coupling analysis, and functional connectivity analysis. Cluster-based permutation tests revealed that theta feedback induced a significant frontal 4–7 Hz suppression cluster (cluster *p* = 0.004), whereas SMR feedback did not produce significant 12–15 Hz enhancement at the group level. Theta feedback elicited cross-frequency spillover as follows: sensorimotor SMR power decreased significantly in theta responders (d = −0.69), with frontal theta and sensorimotor SMR changes positively correlated (r = 0.67, *p* < 0.001). Functional connectivity analysis using debiased weighted phase lag index (dwPLI) further demonstrated significant theta-band network reorganization (cluster *p* = 0.034). At the neural level, clear modulation effects were observed, but shooting ring values did not improve significantly under feedback conditions, and aiming time was significantly prolonged—a behavioral pattern consistent with potential dual-task interference from task-embedded auditory feedback. Single-session auditory NFT can act on the prefrontal cognitive control network and induce cross-frequency network reorganization, but the feedback channel itself constitutes a parallel task that may limit the short-term transfer of induced neural states to behavioral performance. This study examined the neural mechanisms of task-embedded auditory NFT and reported the dual-task costs that have been less characterized in prior “task + feedback” research, providing design considerations and preliminary mechanistic evidence for future development of auditory NFT in precision motor skill training.

## 1. Introduction

Shooting is a precision motor sport that places exceptionally high demands on psychological stability and fine motor control [1,2]. Unlike dynamic sports dominated by explosive power, shooting performance relies critically on the athlete’s capacity to maintain efficient perception–motor integration under low-energy expenditure, a phenomenon referred to as neural efficiency [1,3,4]. Electroencephalographic (EEG) research has demonstrated that expert marksmen exhibit a characteristic pattern of neural oscillations during the pre-shot preparation period: enhanced sensorimotor rhythm (SMR, 12–15 Hz) accompanied by suppressed theta-band (4–7 Hz) activity, particularly reflected in reduced frontal midline theta (FMT) power [5,6,7]. SMR enhancement is generally interpreted as the active gating of irrelevant sensory input by the sensorimotor cortex, facilitating the low-noise state required for motor execution [8], whereas FMT reduction is associated with decreased cognitive control load and efficient allocation of attentional resources, reflecting a shift from explicit control to automatized processing [2,9,10]. This process is often accompanied by an overall reduction in prefrontal functional activity, known as transient hypofrontality, which is considered a key neural marker of high-level motor performance [11,12].

For moderately skilled shooters—individuals who have completed systematic basic training (approximately 30 instructional hours), can independently perform standard unsupported standing shooting, but have not yet competed in formal events—the aforementioned efficient neural oscillatory pattern has not been established. This group possesses standardized foundational shooting skills, yet their neural control patterns have not reached expert levels and still retain substantial plasticity, making them an ideal population for examining the short-term regulatory effects of NFT. At the neural level, moderately skilled shooters typically exhibit elevated cortical activation and reduced information processing efficiency, manifesting a “high-noise, low-efficiency” cortical state [13]. This diffuse cortical activation pattern prevents moderately skilled shooters from spontaneously entering the expert-like “low arousal–high focus” neural state, thereby constraining improvements in shooting performance. Consequently, how to guide moderately skilled shooters to develop goal-directed neural rhythm regulation through external interventions has become a critical scientific question in this field.

Neurofeedback training (NFT) based on brain–computer interfaces (BCIs) provides an effective approach to addressing this issue. NFT maps EEG features onto external feedback signals in real time, enabling individuals to progressively learn to regulate their own neural activity through operant conditioning mechanisms [14,15]. Existing research has confirmed that frequency-band-specific NFT can significantly improve cognitive function and motor performance as follows: SMR enhancement training can improve attentional stability and sensorimotor integration efficiency [16,17], while theta suppression training can reduce prefrontal cognitive load and facilitate automatized processing [16,17]. In competitive sports, NFT has been applied to precision motor events including golf, archery, and shooting, yielding preliminary positive outcomes in optimizing pre-shot neural states [18,19,20].

The application of NFT in the precision motor domain can be broadly categorized into two paradigms. The first is the standalone training paradigm, in which participants receive feedback training in a context separated from the target motor task [19]. The theoretical assumption is that enhancing general cognitive abilities (such as sustained attention, relaxation, and working memory) will enable the acquired capabilities to transfer to the target task. The early literature predominantly adopted this paradigm and has accumulated a body of evidence supporting its training efficacy; however, this approach requires separate training sessions and cannot provide real-time guidance during actual task execution.

The second paradigm, which has emerged in recent years, embeds feedback signals directly into the motor task, forming a “task + feedback” mode. Representative work includes auditory feedback studies targeting golf putting and biathlon shooting [18,21,22], which use the auditory channel to deliver real-time neural state information to guide state adjustment. The potential advantage of this mode lies in leveraging the high temporal resolution of EEG to provide moment-to-moment state cues.

However, embedding feedback within the motor task introduces an aspect that has been less thoroughly examined in the existing literature: dual-task interference. In the task-embedded paradigm, participants must simultaneously complete the primary motor task and monitor auditory feedback. From the perspective of cognitive load theory [23,24], these two information streams may compete for limited central processing resources, a competition that may be particularly pronounced in moderately skilled participants whose motor programs have not yet become automatized [25,26]. Auditory feedback thus provides an external reference for neural state regulation to promote learning on the one hand, while potentially consuming limited cognitive resources and interfering with the ongoing motor task on the other [27,28]. Yet, to date, task-embedded auditory NFT studies have mostly adopted pre-test–post-test comparisons [18,21,22], and, to our knowledge, do not separately report behavioral performance during the feedback period itself, making the dual-task costs that the auditory channel may introduce difficult to detect in these designs. This study therefore treats the task-embedded auditory NFT paradigm as a subject for mechanistic investigation, using a within-subject no-sound baseline control to examine feedback-induced neural changes, cross-frequency transfer, and the manifestation of dual-task interference in behavioral performance during the feedback period.

Despite the promising evidence that NFT can improve sport-related neural states, the existing literature has several key limitations. First, most sport-domain NFT studies employ visual feedback paradigms; however, the visual channel serves the core functions of aiming and target tracking in shooting, and introducing visual feedback may directly interfere with task execution. The auditory channel has been empirically applied as an alternative feedback modality in a few precision motor studies [21], but systematic evidence remains limited. Second, most studies adopt multi-session designs, and direct evidence for single-session immediate effects is scarce [22], yet characterizing single-session effects is essential for understanding NFT mechanisms of action and optimizing training protocols. Third, the majority of studies focus solely on regulation effects in the targeted frequency band, with little systematic examination of cross-frequency spillover effects and whole-brain functional connectivity reorganization induced by NFT. Fourth, most existing analyses rely on region-of-interest (ROI)-level parametric tests and seldom employ whole-brain-scale nonparametric methods such as cluster-based permutation tests, potentially missing distributed neural effects.

Beyond dual-task load concerns, NFT may also induce cross-frequency entrainment effects. Prior research has found that regulation targeting one frequency band may simultaneously affect activity in adjacent or functionally related bands [15,16,17,29]; however, whether different bands exhibit systematic response amplitude differences—particularly whether SMR, closely linked to motor preparation, is significantly affected during theta-suppression-targeted feedback—lacks direct evidence. Clarifying such differentiated cross-frequency response patterns is of both theoretical and practical value for optimizing feedback targeting strategies and avoiding unintended interference with critical motor rhythms.

Furthermore, NFT effects exhibit substantial individual variability. Prior studies have reported that approximately 15–30% of participants cannot establish effective neural regulation within closed-loop paradigms, a phenomenon termed BCI illiteracy [30,31]. This phenomenon is particularly pronounced in single-session designs, as individuals lack sufficient learning trials to establish stable regulation strategies. Therefore, explicitly distinguishing between responders and non-responders in the analysis is methodologically important for revealing the true effects of NFT and its boundary conditions.

Building on the theoretical and practical background outlined above, this study designed and implemented a closed-loop BCI system based on multi-level heartbeat sound feedback. Compared to conventional pure tones or visual feedback, heartbeat sounds represent an interoception-related physiological auditory signal that may possess higher biological familiarity [32,33], providing continuous neural state feedback to participants while maintaining low cognitive load, and thus may be better suited to high-focus task contexts such as shooting. Moderately skilled shooters were selected as the study population for the following two reasons: first, this group already possesses the foundational motor ability to execute standardized shooting tasks, ensuring the experimental task can be completed effectively; second, their neural control patterns are still transitioning from explicit control toward automatized processing, conferring higher sensitivity to external interventions than experts with established neural patterns. This study examined, at the whole-brain scale using cluster-based permutation tests, the immediate effects of single-session auditory NFT on EEG rhythmic activity, whole-brain functional connectivity, and behavioral performance in moderately skilled shooters.

Centered on the core logical chain of “neural regulation—cross-frequency influence—behavioral performance,” this study proposes the following hypotheses:

**H1.**  *Neural Regulation Effect Hypothesis: Under feedback conditions, theta feedback will induce significant suppression of frontal 4–7 Hz power; SMR feedback will induce enhancement of central 12–15 Hz power. Given the different action pathways of the two feedback mechanisms—theta suppression primarily relies on top-down regulation by the prefrontal cognitive control network, while SMR enhancement depends on local oscillatory regulation of the sensorimotor cortex—their group-level effect sizes may differ.*

**H2.**  *Cross-Frequency Coupling and Functional Network Reorganization Hypothesis: Regulation of the targeted frequency band may influence non-targeted band activity through inter-cortical functional coupling. Additionally, NFT may trigger topological reorganization of whole-brain functional connectivity networks, manifested as systematic changes in connection strength or network organization patterns within specific frequency bands.*

**H3.**  *Dual-Task Interference and Neural–Behavioral Dissociation Hypothesis: Because auditory feedback and the shooting task are presented concurrently, the feedback channel itself constitutes a secondary task. It is hypothesized that behavioral performance during the feedback period will not improve significantly relative to the no-sound baseline and may even exhibit behavioral compensation (e.g., prolonged aiming time or spatial accuracy gained at the cost of speed), even when target-band neural activity has been successfully modulated. This neural–behavioral dissociation, potentially attributable in part to dual-task interference, is expected to be particularly pronounced in moderately skilled shooters who have not yet formed automatized motor programs* [34,35].

**H4.**  *Exploratory Hypothesis—Resting-State Offline Effects: Single-session NFT may produce lasting effects on resting-state EEG after training completion, manifested as pre-to-post training changes in target band power or functional connectivity. Given the limitations of the single-session design, the direction and stability of such effects remain uncertain, and this study will examine them in an exploratory manner.*

**H5.**  *Individual Difference Hypothesis: Some participants may fail to establish effective neural regulation within a single session (i.e., non-responders), and responders and non-responders may differ significantly in training effects, functional connectivity patterns, and resting-state baseline characteristics.*

## 2. System Architecture and Core Implementation

**Figure 1:**
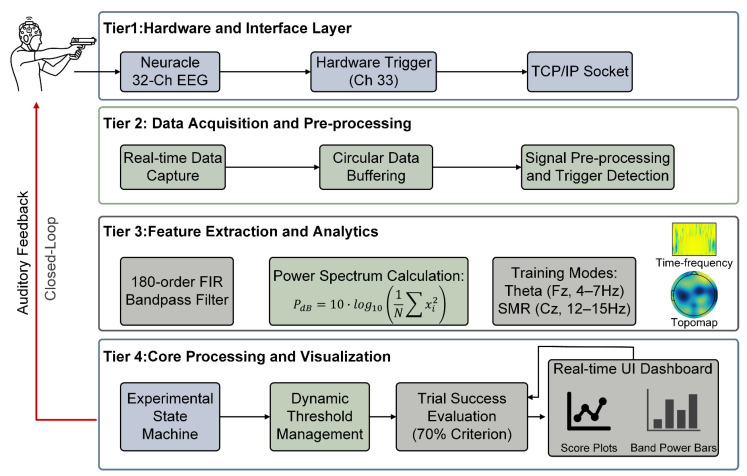
Four-tier architecture of the real-time auditory neurofeedback system. Tier 1: hardware interface; Tier 2: data acquisition and pre-processing; Tier 3: feature extraction; Tier 4: core processing and 5-level heartbeat sound feedback. The red arrow indicates the closed-loop feedback pathway.

This section describes the design and implementation of the closed-loop BCI system based on multi-level heartbeat sound feedback. The system architecture is organized into the following four tiers (see Figure 1): Tier 1 (hardware interface) performs multi-channel EEG signal acquisition and transmission; Tier 2 (data preprocessing) handles signal buffering and denoising; Tier 3 (feature extraction) conducts frequency-domain filtering and power quantification; and Tier 4 (core processing) integrates dynamic threshold management, trial success evaluation, and five-level heartbeat sound feedback, forming a complete closed-loop regulation pathway.

### 2.1. Hardware and Communication Architecture

The system employs a Neuracle 32-channel portable EEG acquisition system as the hardware platform [36,37] and implements a client–server communication architecture based on the TCP/IP protocol (see Figure 1, Tier 1).

The communication management module features the following characteristics: (1) dynamic configuration of server IP address and port to accommodate different LAN deployment environments; (2) a communication link operating at a 1000 Hz sampling rate, fully preserving the phase and frequency information of EEG signals; (3) real-time reception of 33 data streams via a proprietary interface, including 32 standard EEG channels and one hardware synchronization trigger channel for marking the moment of firing; and (4) a built-in 3 s circular buffer that mitigates network jitter effects through asynchronous data acquisition.

### 2.2. Signal Processing Pipeline

The signal processing pipeline corresponds to Tiers 2–3 in Figure 1. The core processing unit uses a 1000 ms window length, with the main timer extracting the most recent 1 s data segment from the circular buffer every 1 s. Both the update step and window length are 1 s, forming a continuous, non-overlapping temporal feature extraction scheme. This design reduces computational load while avoiding over-smoothing and feature delay that overlapping windows may introduce.

Within each processing cycle, the raw signal first undergoes online detrending to remove baseline drift caused by electrode polarization or respiratory motion. Subsequently, a 180th-order finite impulse response (FIR) bandpass filter is applied for frequency-domain selection: theta band (4–7 Hz) is extracted from channel Fz, and SMR band (12–15 Hz) from channel Cz. FIR filters were selected over infinite impulse response (IIR) filters because high-order FIR filters possess strict linear-phase properties that do not introduce phase distortion, which is critical for precise temporal alignment with the firing moment in shooting tasks [38].

Feature quantification employs log-power transformation, converting time-domain signals to decibel (dB) values:

(1) $$P_{\mathrm{dB}}\;=\;10\cdot \log_{10}\;(\frac{1}{N}\cdot \sum {x_{i}}^{2}),\;i=\;1,\;2,\;\ldots ,\;N$$

where *x_i_* denotes the filtered signal sample points and *N* = 1000. The logarithmic transformation helps normalize the distribution of neurophysiological measures and enhances robustness against artifact noise [39], while the dB scale matches the logarithmic perceptual characteristics of human hearing, facilitating the subsequent level-mapped auditory feedback design. The computed real-time feature values are passed as input to the dynamic threshold module (Section 2.3) and the feedback module (Section 2.4).

### 2.3. Dynamic Threshold Adjustment Mechanism

Individual baseline variability in NFT is considerable, and participants are susceptible to fatigue or adaptation effects during prolonged experiments. To address this, the system employs a block-based dynamic threshold adjustment mechanism comprising the following three components: initial threshold setting, trial success determination, and block-level threshold updating.

Initial Threshold Setting. Before formal training, the system computes the mean (μ) and standard deviation (σ) of the target frequency band from individually collected baseline data. Initial thresholds are set leniently: in theta suppression mode, the threshold is set to μ + 0.5σ (making it relatively easy for participants to reduce power below the threshold); in SMR enhancement mode, the threshold is set to μ − 0.5σ (making it relatively easy to raise power above the threshold). This design ensures that participants receive sufficient positive feedback at the start of training to maintain motivation.

Trial Success Determination. After each shot, the system retrospectively extracts the 3 s EEG window preceding the firing event and constructs four overlapping evaluation sub-windows of 1 s length at 500 ms intervals (the last window ends 500 ms before firing to avoid movement artifacts). For each sub-window, the log-power of the target band is computed and compared against the threshold (theta mode requires below-threshold; SMR mode requires above-threshold). A trial is classified as successful when at least two of the four sub-windows meet the criterion (“two-of-four rule”). This redundant evaluation mechanism requires participants to maintain a sustained regulation state before firing, rather than relying on momentary signal fluctuations. The overlapping window design was chosen to provide higher temporal resolution with a 500 ms update step (compared to 1000 ms for a non-overlapping scheme) to more robustly capture the continuity of the pre-shot neural regulation state. A robustness comparison analysis based on non-overlapping windows is provided in Supplementary Table S12. Additionally, a minimum inter-shot interval of 3.0 s is enforced to exclude spurious duplicate trigger events.

Block-Level Threshold Updating. The system adopts a human-in-the-loop adaptive threshold strategy [14,15], using blocks of 10 trials each to dynamically adjust threshold difficulty based on block success rate. When the success rate reaches 70%, threshold updating is triggered as follows: in theta mode, the threshold is lowered (stricter suppression target); in SMR mode, the threshold is raised (stricter enhancement target). The adjustment step size is set by the experimenter based on the participant’s learning progress, maintaining the success rate within the 50–70% optimal zone. Upon threshold updating, the system simultaneously recalculates the five-level feedback boundary points (see Section 2.4).

### 2.4. Multi-Level Heartbeat Sound Feedback Design

#### 2.4.1. Selection of Feedback Carrier

The system employs simulated heartbeat sounds in place of the pure tone feedback conventionally used in NFT. Heartbeat sounds are endogenous physiological rhythm signals that humans begin to perceive from the fetal stage, possessing interoception-related physiological signal characteristics and relatively low attentional intrusiveness [32,33,40]. The system modulates the playback rate and volume intensity of heartbeat sounds to map the participant’s real-time neural state changes, constituting a physiological-metaphor-based auditory feedback approach [41,42,43].

#### 2.4.2. Five-Level Feedback Hierarchy

The system maps neural regulation states into five levels (Level 1–5) based on the relationship between real-time feature values and the dynamic threshold. Centered on the dynamic threshold, the system establishes two boundary points on each side at intervals of 0.5 and 1.0 individual standard deviations (σ), dividing the feature value space into five zones. Level 1 corresponds to the optimal regulation state (highest power in SMR mode, lowest power in theta mode), Level 5 corresponds to the worst state, and Level 3 represents the baseline level near the threshold. Level inversion across the two feedback modes ensures a consistent mapping direction: regardless of mode, Level 1 always represents the optimal state. Boundary points are recalculated whenever the dynamic threshold is updated.

#### 2.4.3. Acoustic Parameters for Each Level

The acoustic parameters for the five levels are presented in Table 1. The design follows the principle of “better state, quieter feedback”: Level 1 outputs an extremely soft, slow single heartbeat sound, minimizing interference with the shooting task. As the regulation state deviates from the target direction, volume increases and playback rate accelerates progressively. Level 4 features a double heartbeat with an 80 ms interval, and Level 5 features a triple heartbeat with a 60 ms interval, simulating the perceptual characteristics of heart rate acceleration under stress.

#### 2.4.4. Fade-In/Fade-Out and Guard Band Mechanisms

The system applies linear amplitude fade-in/fade-out processing at the audio output stage (duration parameters shown in Table 1), eliminating transient noise produced by the abrupt onset or offset of audio segments.

Additionally, a guard band logic is introduced to prevent frequent feedback-level switching caused by minor signal fluctuations. When the feature value falls within the Level 3 zone (threshold ± 0.5 × σ) and the current level is Level 2–4, the system maintains the current level; only when the level is at Level 1 or Level 5 does the system adjust stepwise (±1 per step) toward the target level.

**Figure 2:**
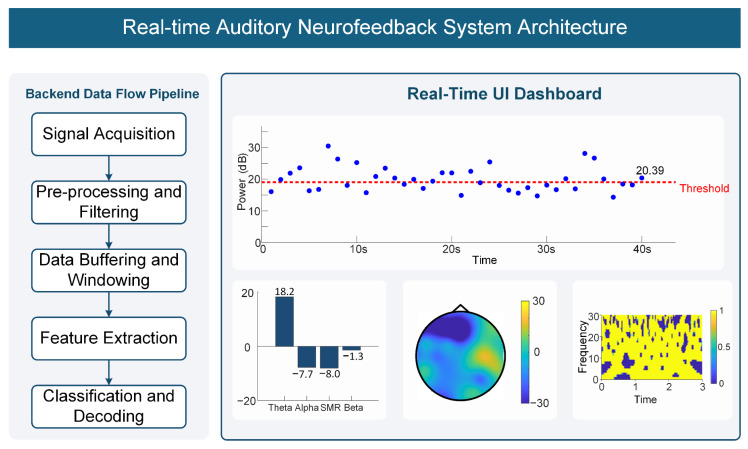
Real-time system user interface. **Left panel**: backend data flow pipeline including signal acquisition, preprocessing, buffering, feature extraction, and classification. **Right panel**: real-time UI dashboard showing feature value trend plot with dynamic threshold (red dashed line), band power bar chart, topographic map, and time–frequency spectrogram.

The system user interface (see Figure 2) provides real-time feature value trend plots, frequency band power bar charts, topographic maps, and time–frequency spectrograms for the experimenter to monitor participant neural states in real time.

## 3. Materials and Methods

### 3.1. Participants

Twenty-two university students (20 males, 2 females; aged 19–24 years, M = 21.3, SD = 1.2) were recruited. All participants were right-handed with normal or corrected-to-normal vision, normal hearing, no history of epilepsy, had not taken medications affecting the central nervous system within the two weeks preceding the experiment, had abstained from caffeine or alcohol for 24 h prior to testing, and had slept at least 7 h the night before.

Regarding shooting experience, all participants had completed the basic shooting elective course at their university (approximately 30 instructional hours), could independently perform standard unsupported standing shooting with passing grades or above, but had not participated in formal competitions and had not yet formed stable automatized shooting skills. This experience level was selected because such individuals possess the foundational motor ability to execute standardized shooting tasks while their neural control patterns still retain substantial plasticity [4,13,44].

Sample size was determined by reference to the typical scale of sport performance-oriented EEG–NFT studies (15–25 participants) [45,46], accounting for the approximately 15–30% non-responder rate [31,47] to ensure the responder subgroup retained at least 9 participants for effective analysis. Post hoc power analysis (noncentral t-distribution method) indicated that with *N* = 21 in a paired design (two-tailed α = 0.05), the study had adequate detection power for large effect sizes (d = 0.80, 1 − β = 0.94) but limited sensitivity for medium effect sizes (d = 0.50 (see Table S1 for the full power analysis reference table), 1 − β = 0.59); this limitation is addressed further in the Section 5. This study adhered to the provisions of the Declaration of Helsinki. The study was approved by the Ethics Committee of the Engineering University of the Chinese People’s Armed Police Force (approval date: 13 March 2025). All participants provided written informed consent.

### 3.2. Experimental Equipment and Shooting Task

The experiment employed an optical shooting simulation system comprising a laser-based simulated pistol and an accompanying optical target detection device. The system captures the two-dimensional coordinates of each impact point in real time through the optical sensor head at the muzzle aligned with a reflective target surface [48,49]; shooting scores (ring values) were automatically recorded and exported by the accompanying commercial software. Each shot record includes phase number and sequence markers, ensuring precise temporal alignment between behavioral and EEG data.

Considering physical space constraints of the experimental venue, the standard shooting environment was proportionally scaled as follows: shooting distance was shortened from 25 m to 10 m, and the standard chest ring target (50 × 50 cm) was correspondingly reduced to 20 × 20 cm. This scheme preserved aiming angle consistency (standard condition: 2 × arctan(25/2500) ≈ 1.146°; scaled condition: 2 × arctan(10/1000) ≈ 1.146°), maintaining equivalent aiming precision demands [50]. Environmental controls included: room temperature 22–25 °C, closed doors and windows to isolate external noise, and constant illumination. Participants adopted the standard unsupported standing position [51,52].

### 3.3. Experimental Procedure

**Figure 3:**
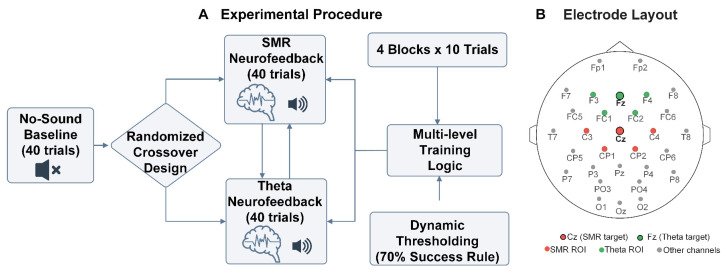
Experimental protocol and electrode configuration (within-subject design). All 22 participants completed all three task conditions in a single session. The no-sound baseline was always presented first; the order of SMR enhancement and theta suppression conditions was counterbalanced across participants (randomized crossover). (**A**) Temporal sequence of experimental phases with adaptive feedback logic (right inset). (**B**) 32-channel EEG electrode layout. Target electrodes are highlighted as larger filled circles with black borders (red: Cz, SMR target; green: Fz, Theta target). SMR ROI channels (C3, C4, CP1, CP2) are shown as red filled circles; Theta ROI channels (F3, F4, FC1, FC2) as green filled circles; all other channels in gray (see in-figure legend).

The experiment was designed to evaluate the immediate effects of single-session auditory NFT on EEG rhythms and behavioral performance in moderately skilled shooters. Total duration was approximately 90–120 min, divided into four phases (see Figure 3A).

Phase 1: Resting-State Baseline Acquisition. After donning the EEG cap, participants sequentially underwent 1.5 min eyes-open and 1.5 min eyes-closed resting-state EEG recording, serving as the pre-training neural baseline [53,54].

Phase 2: Pre-Experimental Calibration. Participants completed approximately 10–15 shots without feedback, from which the system computed individual baseline mean and standard deviation for the target frequency band and set the initial feedback threshold (threshold calculation and dynamic adjustment mechanism detailed in Section 2.3).

Phase 3: Core Experiment (Three Task Conditions). Each condition comprised 40 shots divided into 4 blocks of 10. The three conditions were administered sequentially as follows: (1) No-Sound Baseline (No_Sound_FB): no auditory feedback, serving as the behavioral and EEG baseline control, always presented first to avoid carryover effects from feedback training; (2) SMR Enhancement (SMR_FB): training target was to increase Cz channel 12–15 Hz power; and (3) Theta Suppression (Theta_FB): training target was to decrease Fz channel 4–7 Hz power. The presentation order of SMR_FB and Theta_FB was counterbalanced across participants using randomized crossover [53], with 2–3 min rest between conditions.

Feedback sounds were delivered via in-ear earphones at a comfortable volume. Before the experiment, participants received approximately 5 min of standardized instructions informing them that “the quieter and slower the heartbeat sound, the closer the current neural state is to the ideal focused state,” without disclosing specific frequency band information or regulation strategies to avoid cognitive expectancy interference [18,55]. All three conditions used self-paced triggering, with participants deciding firing timing based on feedback cues or their own state assessment [11].

Phase 4: Post-Training Resting-State Acquisition. Following training completion, 1.5 min eyes-open and 1.5 min eyes-closed resting-state EEG data were again recorded for offline effect evaluation.

### 3.4. EEG Signal Acquisition and Preprocessing

EEG was recorded using a Neuracle 32-channel portable system [36,37]. Electrode placement followed the extended international 10–20 system, covering frontal, central, parietal, occipital, and temporal regions (complete montage shown in Figure 3B). The online reference was placed on the midline between Cz and Pz; the ground electrode was placed on the anterior midline frontal area. Sampling rate was 1000 Hz with online bandpass filtering of 0.01–200 Hz. All electrode impedances were kept below 5 kΩ using conductive gel. Channel 33 served as the hardware trigger for real-time marking of firing events, achieving millisecond-level temporal alignment between EEG and behavioral data.

Offline preprocessing was performed in MATLAB R2025b using the EEGLAB toolbox [56]. Raw continuous EEG was first stripped of non-brain channels (Trigger, Annotation, ECG, VEO, and HEO), then bandpass-filtered at 1–45 Hz (Hamming-windowed FIR filter) with a 49–51 Hz notch filter to remove power-line interference [38]. Task-state data were epoched from −6.0 s to 0 s relative to the firing trigger; duplicate trigger marks within 1 s of the same event were reduced to the first occurrence to ensure segmentation accuracy. Resting-state data were segmented into continuous non-overlapping 2 s epochs. No baseline correction was applied to task-state data to preserve absolute power-level information of pre-shot neural oscillations.

Artifact removal proceeded in two steps. First, a joint probability method was applied as an initial screen, rejecting trials whose joint probability exceeded 8 standard deviations [57]. Second, independent component analysis (ICA) decomposition was performed on the remaining data, with PCA dimensionality automatically set to the actual rank of the data matrix. ICLabel [58] was used to automatically classify each component, and non-brain-source components (ocular, muscular, cardiac, etc.) with classification probability exceeding 0.9 were removed [59]. Automated bad-channel detection and interpolation were not performed to avoid introducing spurious signals through spatial reconstruction. Participants whose valid trial retention rate fell below 80% in any condition were excluded. Finally, whole-brain average re-referencing was applied to the retained data [60]. Data quality diagnostics for all participants are provided in Figure S1.

### 3.5. Feature Extraction

#### 3.5.1. Task-State Time–Frequency Analysis

Time–frequency analysis was performed using the FieldTrip toolbox [61], employing Morlet continuous wavelet transform (width parameter: 5 cycles) over a frequency range of 4–45 Hz (1 Hz steps) with 50 ms temporal resolution [62]. Power spectra were expressed in decibels (dB = 10·log_10_(power); formula in Section 2.2) to compress magnitude differences. The time windows of interest for statistical analysis were set according to frequency band characteristics as follows: for cluster-based permutation tests, the SMR analysis window was −5.5 s to −0.5 s pre-shot and the theta window was −5.0 s to −1.0 s [7]. For ROI validation analyses, the SMR time window was −5.0 s to −0.5 s and the theta window was −5.0 s to −1.0 s. The trailing edges of both windows avoid the firing moment to exclude movement artifacts, and the leading edges allow sufficient margin to mitigate edge effects of the wavelet transform [8,62].

#### 3.5.2. Task-State Functional Connectivity and Network Metrics

Functional connectivity strength between channel pairs was quantified using the debiased weighted phase lag index (dwPLI) [63], which is computed from the imaginary part of the cross-power spectrum and is robust to volume conduction effects. To further suppress volume conduction and common-reference artifacts, scalp current density (SCD) transformation (spline interpolation method, finite difference method as fallback, and raw data in rare failure cases) was applied before computing functional connectivity.

For each participant and condition, complex Fourier spectra were first extracted using multitaper fast Fourier transform (mtmfft, frequency smoothing parameter tapsmofrq = 1 Hz) over a frequency range of 4–30 Hz. Full-channel dwPLI matrices (32 × 32) were then computed for both the theta and SMR bands, with global connectivity strength defined as the mean of upper-triangular elements. Additionally, the Fz–Cz channel-pair dwPLI was extracted as an a priori ROI indicator of frontal–central regional connectivity.

For network topology analysis, individual dwPLI matrices were binarized using a proportional threshold (retaining the top 20% of connection strengths), and global efficiency, clustering coefficient, weighted strength, and degree heterogeneity were computed [64]. Note that the A1 and A2 mastoid electrodes were included in all statistical analyses (yielding a 32 × 32 connectivity matrix). For topographic visualization purposes, edges involving A1 and A2 are omitted from the scalp network plots because these electrodes lack standard scalp positions in the 2-D projection.

#### 3.5.3. Resting-State Feature Extraction

Pre- and post-training resting-state EEG data were extracted. Resting-state power analysis employed Hanning-windowed fast Fourier transform (FFT) over a frequency range of 1–45 Hz, with power reported in dB. Resting-state functional connectivity analysis followed the same SCD + dwPLI pipeline as the task state, with corresponding frequency-band-specific global functional connectivity strength and graph-theoretic metrics extracted.

### 3.6. Statistical Analysis

The significance level for all statistical tests was set at α = 0.05 (unless otherwise noted), with effect sizes reported as Cohen’s d (paired comparisons) or partial η^2^ (ANOVAs), interpreted following Cohen (1988) [65]. Although real-time feedback monitored only the Cz and Fz channels, cortical effects of NFT may not be restricted to target electrodes. Therefore, this study adopted a dual strategy of “whole-brain discovery + ROI validation” as follows: first conducting unbiased data-driven cluster tests across all 32 channels, then performing confirmatory analyses on predefined ROIs, balancing discovery power and hypothesis directedness.

#### 3.6.1. Cluster-Based Permutation Tests

Whole-brain statistical inference employed nonparametric cluster-based permutation tests [66,67]. For each feedback condition, paired comparisons between the feedback and no-sound baseline conditions were conducted within the corresponding responder subgroup (SMR effects tested within SMR responders; theta effects within theta responders). Paired *t*-statistics were computed at each channel–frequency–time point; adjacent significant points exceeding the cluster formation threshold (α_cluster = 0.05) were aggregated into clusters based on Euclidean distance-based spatial adjacency (FieldTrip default distance method). The summed t-value within each cluster served as the test statistic, and corrected *p*-values were computed by constructing a null distribution via Monte Carlo simulation (5000 permutations) [68]. For two-tailed tests, the one-tailed significance threshold was set at α/2 = 0.025.

#### 3.6.2. ROI Validation Analysis

Two ROIs were predefined and mean log_10_ power was extracted within the following corresponding time–frequency windows: SMR_Key_Area (Cz, C3, C4, CP1, and CP2; 12–15 Hz) reflecting sensorimotor integration [8]; Theta_Frontal (Fz, F3, F4, FC1, and FC2; 4–7 Hz) reflecting cognitive control [7]. Paired *t*-tests with Cohen’s d were conducted for each ROI. ROI analyses represent hypothesis-driven confirmatory tests intended to validate and quantitatively describe whole-brain cluster effects; no inter-ROI multiple comparison correction was applied. Consistency verification across different time windows (full window, early segment, and late segment) was also performed.

#### 3.6.3. Functional Connectivity Statistical Analysis

At the macroscopic level, repeated-measure ANOVA (RM-ANOVA) was used to compare conditions, with paired *t*-tests for post hoc pairwise comparisons. At the edge level, custom cluster-based permutation tests controlled for multiple comparisons as follows: paired *t*-tests on each edge identified initially significant edges (*p* < 0.05), which were aggregated into clusters based on shared nodes, with summed within-cluster t-values as the test statistic; corrected *p*-values were derived from a null distribution constructed via 5000 permutations. Subgroup analyses repeated these tests separately within responders and non-responders.

#### 3.6.4. Behavioral and Subjective Evaluation Analysis

Shooting behavior was comprehensively assessed using the following metrics: mean ring value (Hit, primary accuracy index), aiming time (onTarget), horizontal deviation (devX), vertical deviation (devY), trigger quality rating (TIRE, 1/2/3 discrete ordinal variable), relative trigger value (RTV), and aiming curve center-of-gravity mean (COG_Hit) [7]. One-way RM-ANOVAs were conducted for continuous indicators with feedback condition as the within-subject factor; Friedman tests were used for TIRE. Responder and non-responder subgroup analyses further compared each feedback condition against the no-sound baseline using paired *t*-tests (continuous variables) or Wilcoxon signed-rank tests (TIRE).

Subjective evaluation comprised the following three questionnaire categories: (1) Mini Questionnaire (MiniQ): completed after each block, rating four dimensions on a 7-point Likert scale; (2) Mode Questionnaire (ModeQ): completed post-training, covering six dimensions; and (3) Overall Questionnaire (OverallQ): mode preference distribution and training satisfaction. Statistical tests included Wilcoxon signed-rank tests, Friedman tests, and binomial tests for preference distributions. Presentation order effects were examined using Mann–Whitney U tests.

#### 3.6.5. Responder Definition

Responders were operationally defined based on single-electrode metrics consistent with the real-time feedback system (primary definition) as follows: a participant was classified as an SMR responder if Cz channel 12–15 Hz log_10_ power under SMR_FB was higher than under No_Sound_FB; a participant was classified as a Theta responder if Fz channel 4–7 Hz log_10_ power under Theta_FB was lower than under No_Sound_FB. Power extraction used a time window of −3.0 s to −0.5 s. Specifically, for each participant, the power spectrum was first averaged across all valid trials during the time–frequency analysis stage (Morlet wavelet → cross-trial mean), then log_10_-transformed, and finally the mean log_10_ power in the target frequency band (12–15 Hz or 4–7 Hz) and time window (−3.0 s to −0.5 s) at the target electrode (Cz or Fz) was extracted as the scalar metric for responder classification. As a robustness check, all subgroup analyses were repeated using target-band ROI means as an auxiliary definition (Cohen’s κ assessed inter-definition agreement; full results in Table S2).

#### 3.6.6. Resting-State Pre–Post Analysis

Pre- and post-training resting-state EEG was extracted (approximately 1 min each for eyes-open and eyes-closed segments). Power analysis used Hanning-windowed FFT (1–45 Hz, dB units). Fz theta power and Cz SMR power served as primary indicators; Central ROI (C3, Cz, and C4) SMR and Frontal ROI (F3, Fz, and F4) theta served as supplementary validation indicators. Paired *t*-tests (pre vs. post) were used for pre–post comparisons [14]. One participant (Subject 01) was excluded due to missing post-test resting-state data, yielding a final sample of *N* = 21. Resting-state functional connectivity analysis followed the same SCD + dwPLI pipeline, with edge-level cluster-based permutation tests (5000 permutations) controlling for multiple comparisons.

#### 3.6.7. Responder Characteristic Comparison

To explore neurophysiological differences between responders and non-responders, group comparisons were conducted along the following three dimensions: (1) pre-training resting-state baseline (channel-wise band power + global FC strength); (2) resting-state pre–post change; and (3) task-state FC. Independent-sample *t*-tests were used as the primary analysis, with Mann–Whitney U tests reported as robustness references when either subgroup had *N* < 10. Channel-/edge-level multiple comparisons were controlled using Benjamini–Hochberg FDR correction.

#### 3.6.8. Outlier Sensitivity Analysis

Outlier sensitivity analyses were performed on all reported statistical tests following a multiverse analysis approach [69]. Outlier detection employed the following three complementary methods: interquartile range (IQR × 1.5), median absolute deviation (MAD × 3), and Grubbs test (α = 0.05). Each test was repeated on original data, outlier-excluded data, and Winsorized data, and the number of tests with changed conclusions was reported. Cluster-based permutation tests, being inherently robust to outliers, were excluded from sensitivity re-analysis. Full results are provided in Table S3 and Figure S2.

#### 3.6.9. Window Overlap Robustness Analysis

The trial success determination (Section 2.3) employs the “two-of-four” rule with four overlapping sub-windows, where adjacent windows share 500 ms of data. To verify that this overlap does not introduce artificial inflation of success rates, we recalculated trial success rates using three non-overlapping 1 s sub-windows (−3.0 to −2.0 s, −2.0 to −1.0 s, and −1.0 to 0 s) under a “two-of-three” rule, and compared them with the original scheme at the same threshold on a trial-by-trial basis. The overlapping scheme yielded slightly higher success rates than the non-overlapping scheme (SMR: +6.5 ± 5.7%; Theta: +4.8 ± 5.0%), but this difference was absorbed in real time by the dynamic threshold adjustment mechanism (Section 2.3), and trial success rates do not enter the offline statistical analysis pipeline of this study (all core conclusions are based on direct statistical tests of EEG power spectra and functional connectivity). Complete per-participant, per-block comparison data are provided in Table S12.

## 4. Results

After artifact rejection, the mean valid trial retention rate exceeded 95% across all conditions for all 22 participants. Following the operational definitions in Section 3.6.5, nien participants (40.9%) were classified as SMR responders and 13 (59.1%) as Theta responders. Subsequent analyses report both full-group and responder subgroup results.

### 4.1. Task-State Neural Modulation Effects

**Figure 4:**
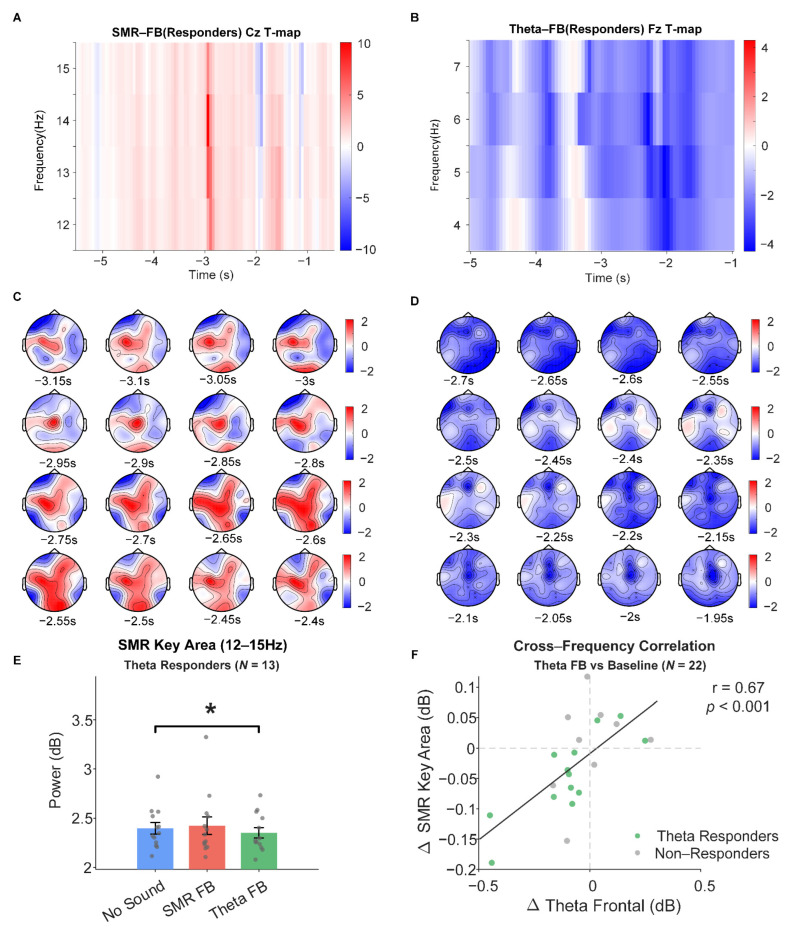
Task-state neural modulation effects and cross-frequency coupling analysis. (**A**) Time–frequency T-map at target electrode Cz for SMR responders (*N* = 9); no significant positive cluster *p* > 0.05). (**B**) Time–frequency T-map at target electrode Fz for Theta responders (*N* = 13); black contour delineates a significant negative cluster (*p* = 0.004). (**C**) Topographic map sequence of SMR-band T-values during the significant time window under SMR feedback in responders. (**D**) Topographic map sequence of Theta power suppression dynamics under Theta feedback in responders. (**E**) Bar plot of SMR Key Area power across three conditions for Theta responders (*N* = 13); * *p* < 0.05. (**F**) Scatter plot of cross-frequency correlation between ΔTheta_Frontal and ΔSMR_Key_Area (*N* = 22); r = 0.67, *p* < 0.001. Colorbar units: T-values.

SMR Feedback Condition. Among SMR responders, the cluster-based permutation test did not identify a significant positive cluster in the SMR band (12–15 Hz). The positive power enhancement trend over the sensorimotor area did not reach significance (*p* > 0.05; see Figure 4A,C). Although SMR responders showed individual-level power enhancement trends (by definition), the small sample size (*N* = 9) and inter-individual variability in the spatiotemporal distribution of effects prevented group-level significance under the stringent cluster permutation test. ROI analysis also showed that SMR power enhancement in the sensorimotor key area did not reach significance in SMR responders (t(8) = 1.50, *p* = 0.172, d = 0.50).

Theta Feedback Condition. Theta responders exhibited a significant power suppression cluster in the theta band (4–7 Hz), concentrated in the approximately −2.2 s to −1.6 s time window, reaching maximum effect at frontal electrodes (e.g., near F3; see Figure 4B,D). Full cluster statistics are reported in Table S4.

ROI Validation and Cross-Frequency Coupling Effects. Among Theta responders, SMR power in the sensorimotor key area (C3, C4, Cz, CP1, and CP2; 12–15 Hz) was significantly lower under theta feedback compared to baseline (t(12) = −2.48, *p* = 0.029, d = −0.69; see Figure 4E), whereas SMR responders showed no significant change under SMR feedback (*p* = 0.293). Pearson correlation analysis of individual-level power changes across the full group (*N* = 22) revealed that frontal theta power change (ΔTheta_Frontal) under theta feedback was significantly positively correlated with sensorimotor SMR power change (ΔSMR_Key_Area) (r = 0.67, *p* < 0.001, 95% CI [0.35, 0.85]; see Figure 4F); Spearman rank correlation was consistent (ρ = 0.71, *p* < 0.001). This correlation was even stronger within the Theta responder subgroup (r = 0.82, *p* < 0.001) and non-significant in non-responders (r = 0.35, *p* = 0.354). Complete ROI statistical results and cross-frequency correlation details are provided in Table S5.

### 4.2. Task-State Functional Connectivity and Whole-Brain Network Analysis

**Figure 5:**
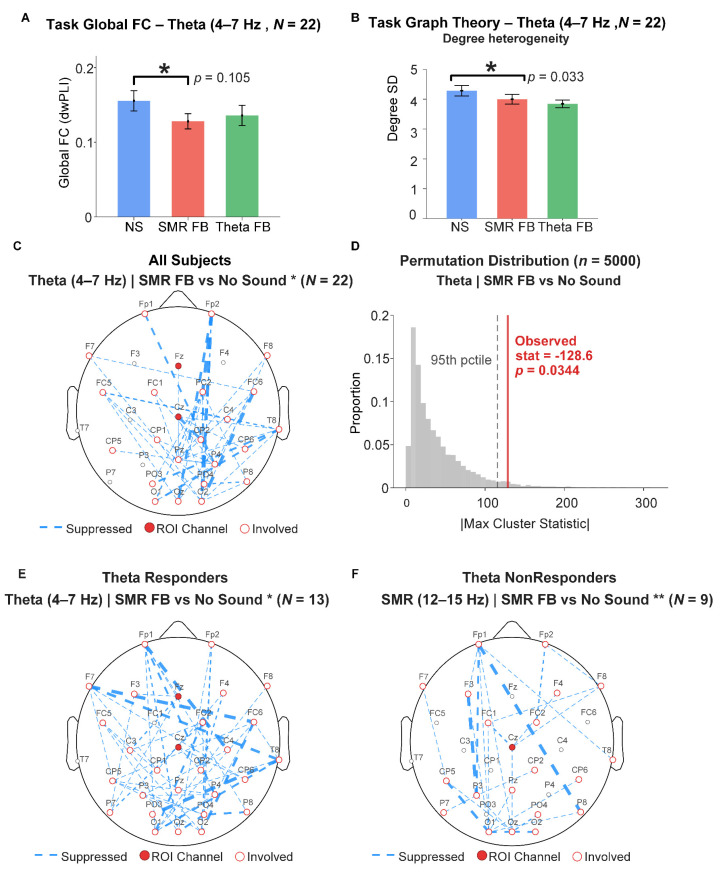
Task-state functional connectivity and network topology analysis. (**A**) Global functional connectivity strength (mean dwPLI) in the theta band across three conditions (*N* = 22); * *p* < 0.05. (**B**) Degree heterogeneity in the theta band across conditions (*N* = 22); * *p* < 0.05. (**C**) Edge-level network visualization of the significant negative cluster (theta band, SMR feedback vs. no-sound baseline, *N* = 22; cluster statistic = −128.6, *p* = 0.034, corrected). (**D**) Permutation distribution (5000 permutations) of the maximum cluster statistic. (**E**) Edge-level network for Theta responders (*N* = 13; cluster statistic = −134.52, *p* = 0.024). (**F**) Edge-level network for Theta non-responders (*N* = 9; SMR band; cluster statistic = −88.34, *p* = 0.003). In the network topographic plots (**C**,**E**,**F**): blue dashed lines indicate connections that were significantly suppressed under feedback relative to the no-sound baseline; line thickness is proportional to the magnitude of the edge-level *t*-statistic (thicker lines represent stronger suppression). Filled red circles denote ROI channels (Fz for theta band, Cz for SMR band); open red circles denote channels that participated in at least one significant connection (“Involved”); small white-filled circles denote channels not involved in any significant connection (“Not involved”). Edges involving the mastoid reference electrodes (A1, A2) are omitted from the topographic plots; all reported statistics are based on the full 32-channel connectivity matrix. Error bars: SEM; * *p* < 0.05, ** *p* < 0.01.

Global Functional Connectivity Strength. In the theta band (4–7 Hz), the condition main effect showed a marginal trend, F(2, 42) = 2.379, *p* = 0.105. Planned pairwise comparisons revealed that global theta-band connectivity was significantly lower under SMR feedback than under the no-sound baseline (t(21) = −2.125, *p* = 0.046, d = −0.45; see Figure 5A), while the difference between theta feedback and baseline was non-significant (*p* = 0.122). The condition main effect in the SMR band (12–15 Hz) was non-significant, F(2, 42) = 1.180, *p* = 0.317.

Network Topology. Degree heterogeneity showed a significant condition main effect in the theta band (F(2, 42) = 3.717, *p* = 0.033). Pairwise comparisons indicated that theta feedback significantly reduced degree heterogeneity relative to baseline (t(21) = −2.685, *p* = 0.014, d = −0.57), suggesting that feedback training shifted the theta-band network toward a more homogeneous topology (see Figure 5B). Global efficiency, clustering coefficient, and weighted strength showed non-significant condition main effects across both frequency bands (*ps* > 0.10; Table S6).

Network-Level Cluster Permutation Tests. In the full sample (*N* = 22), the theta-band comparison of SMR feedback versus no-sound baseline yielded a significant negative cluster (cluster statistic = −128.60, *p* = 0.034, corrected), encompassing 51 edges and 26 channels, covering widespread frontal, central, and parieto-occipital connections (see Figure 5C,D). This indicates that SMR feedback training induced broad reductions in theta-band functional connectivity across the whole brain. All other comparisons and all SMR-band comparisons did not yield significant clusters. Detailed FC matrices, difference matrices, and network topology visualizations are provided in Figure S3.

Subgroup Analysis. In the Theta responder group (n = 13), the theta-band SMR feedback vs. baseline comparison yielded a larger and more significant negative cluster (cluster statistic = −134.52, *p* = 0.024, corrected, 51 edges, 29 channels; see Figure 5E), indicating that the whole-sample theta-band connectivity reduction was primarily driven by Theta responders.

In the Theta non-responder group (*n* = 9), the following two highly significant negative clusters emerged in the SMR band: SMR feedback vs. baseline (cluster statistic = −88.34, *p* = 0.003, 31 edges, 24 channels) and Theta feedback vs. baseline (cluster statistic = −89.24, *p* = 0.010, 31 edges, 24 channels; see Figure 5F). Although this subgroup could not effectively regulate theta power, they exhibited strong connectivity reorganization effects in the SMR band, with both feedback conditions inducing comparable connectivity reductions.

In the SMR non-responder group (*n* = 13), the SMR-band Theta feedback vs. baseline comparison yielded a significant negative cluster (cluster statistic = −69.96, *p* = 0.030, 26 edges, 22 channels), reflecting cross-frequency connectivity modulation. The SMR responder group (*n* = 9) did not produce significant clusters.

### 4.3. Behavioral Performance

**Figure 6:**
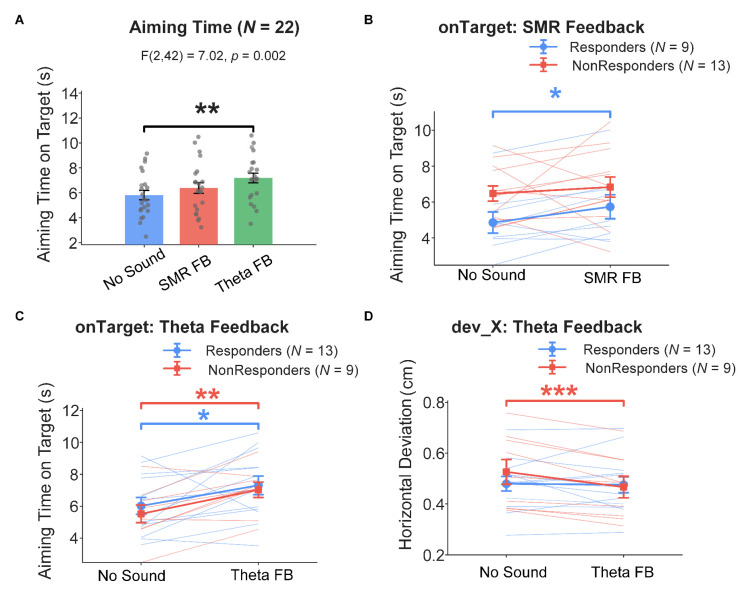
Behavioral performance across feedback conditions and individual difference patterns (*N* = 22). (**A**) Group-level aiming time across three conditions; ** *p* < 0.01. (**B**) Individual trajectories of aiming time under SMR feedback for responders (blue, *N* = 9) and non-responders (red, *N* = 13); * *p* < 0.05. (**C**) Individual trajectories under Theta feedback; both responders (* *p* < 0.05) and non-responders (** *p* < 0.01) showed significant increases. (**D**) Individual trajectories of horizontal deviation under Theta feedback; non-responders showed significant reduction (*** *p* < 0.001, d = −1.75). Error bars: SEM; thin lines: individual participants.

Aiming time (onTarget) showed a significant condition main effect (F(2, 42) = 7.020, *p* = 0.002; see Figure 6A). Subgroup analysis revealed that under SMR feedback, SMR responders’ aiming time was significantly prolonged compared to baseline (t(8) = 2.849, *p* = 0.022, d = 0.95), while non-responders showed no significant change (*p* = 0.565; see Figure 6B). Under theta feedback, both responders (t(12) = 2.230, *p* = 0.046, d = 0.62) and non-responders (t(8) = 3.706, *p* = 0.006, d = 1.24) exhibited significant aiming time prolongation (see Figure 6C). Additionally, the condition main effect for horizontal aiming deviation (dev_X) did not reach significance (F(2, 42) = 2.854, *p* = 0.069), but subgroup analysis revealed that non-responders under theta feedback showed significantly reduced horizontal deviation (t(8) = −5.258, *p* < 0.001, d = −1.75), while responders under SMR feedback showed a marginal trend (t(8) = 2.192, *p* = 0.060; see Figure 6D). Shooting ring value (Hit) showed no significant changes at the group level or in any subgroup (F(2, 42) = 1.912, *p* = 0.160; subgroup paired comparisons *ps* > 0.22). Remaining indicators (dev_Y, TIRE, RTV, and COG_Hit) showed non-significant condition main effects and subgroup comparisons (Table S7, Figure S4).

### 4.4. Resting-State Change Analysis

Paired *t*-tests were conducted on pre- and post-training resting-state EEG data (*N* = 21; Subject 01 excluded due to missing post-test data).

**Figure 7:**
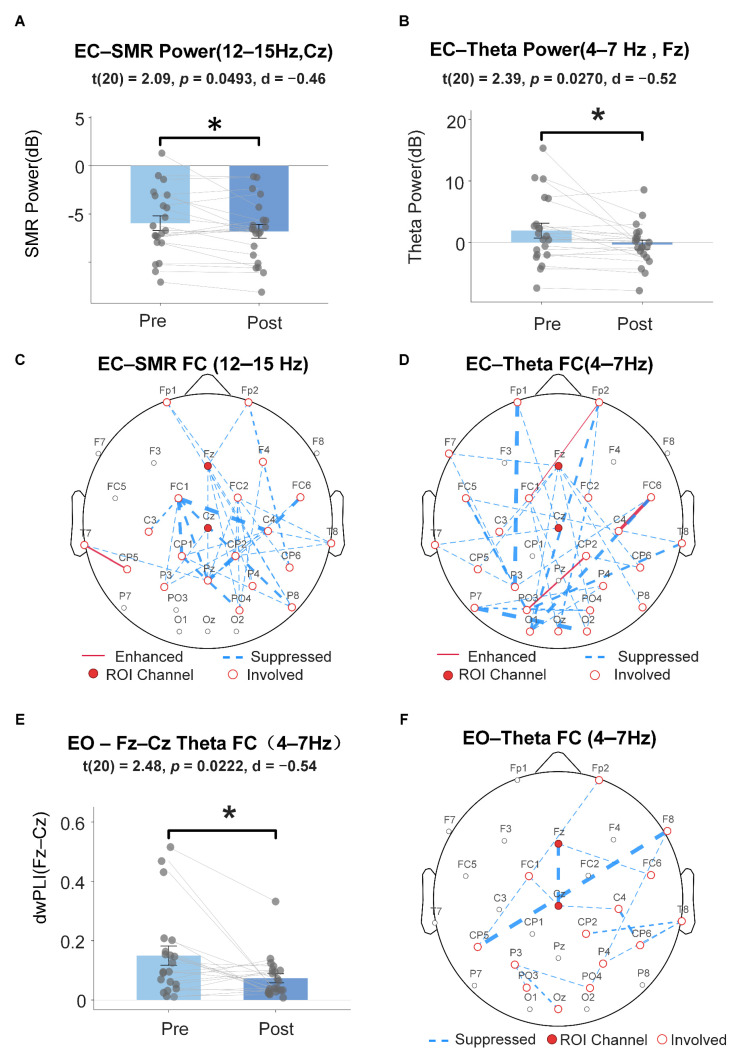
Resting-state neural changes following neurofeedback training (*N* = 21). (**A**) Eyes-closed SMR power (12–15 Hz) at Cz significantly decreased post-training; * *p* < 0.05. (**B**) Eyes-closed theta power (4–7 Hz) at Fz significantly decreased post-training; * *p* < 0.05. (**C**,**D**) Edge-level functional connectivity changes (dwPLI, *p* < 0.05, uncorrected) in the eyes-closed condition for SMR and theta bands. (**E**) Eyes-open Fz–Cz theta-band dwPLI decreased post-training; * *p* < 0.05. (**F**) Edge-level connectivity changes in the eyes-open theta band (14 edges, uncorrected). In the network topographic plots (**C**,**D**,**F**): red solid lines indicate connections that were significantly enhanced post-training (Post > Pre); blue dashed lines indicate connections that were significantly suppressed post-training (Post < Pre); line thickness is proportional to the magnitude of the edge-level *t*-statistic. Filled red circles denote ROI channels (Fz, Cz); open red circles denote channels involved in at least one significant connection (“Involved”); small white-filled circles denote channels not involved (“Not involved”). Edges involving the mastoid reference electrodes (A1, A2) are omitted from the topographic plots; all reported statistics are based on the full 32-channel connectivity matrix.

Band Power. Under the eyes-closed (ECs) condition, post-training Cz SMR power was significantly reduced (t(20) = −2.09, *p* = 0.049, d = −0.46) and Fz theta power was likewise significantly reduced (t(20) = −2.39, *p* = 0.027, d = −0.52; see Figure 7A,B). ROI validation showed concordant reduction trends for Central ROI SMR (*p* = 0.064, d = −0.43) and Frontal ROI theta (*p* = 0.060, d = −0.44). No significant changes were observed under the eyes-open (EOs) condition (*ps* > 0.08; Table S8, Figure S5).

Functional Connectivity. Whole-brain mean dwPLI showed no significant changes in either frequency band under either condition (*ps* > 0.09). Network-based cluster permutation tests (5000 permutations) did not identify significant clusters in any band or condition. However, exploratory edge-level analysis revealed that under the EC condition, 37 edges in the SMR band and 34 edges in the theta band showed changes at uncorrected level (*p* < 0.05). Of these, 33 per band involved scalp electrodes only and are displayed in Figure 7C,D, with the remainder involving the A1/A2 mastoid channels; the scalp-level changes were primarily distributed over central–frontal and central–parieto-occipital regions. The Fz–Cz electrode pair showed significantly reduced theta-band dwPLI under the EO condition (t(20) = −2.48, *p* = 0.022, d = −0.54; Figure 7E), while no significant change was observed in the SMR band (*p* = 0.154). Graph-theoretic metrics showed no significant changes under any condition (*ps* > 0.05; Table S9).

### 4.5. Individual Differences and Subjective Evaluation

**Figure 8:**
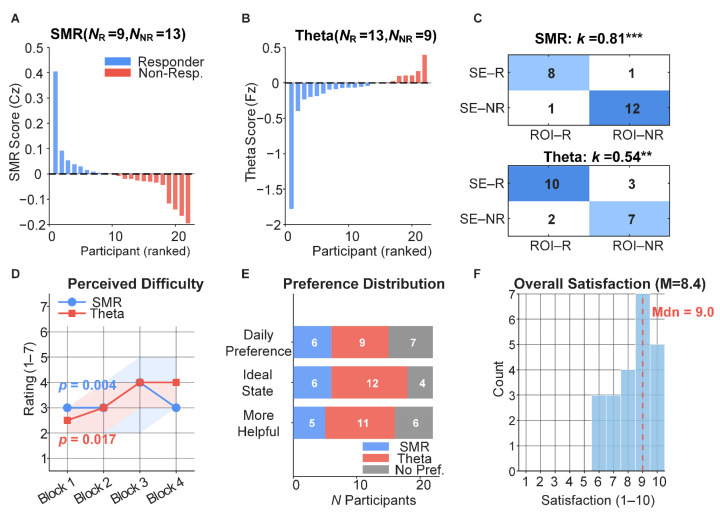
Individual differences in regulation capacity and subjective evaluation. (**A**) SMR regulation scores ranked by magnitude (blue: responders, *N* = 9; red: non-responders, *N* = 13). (**B**) Theta regulation scores ranked by magnitude (blue: responders, *N* = 13; red: non-responders, *N* = 9). (**C**) Cross-classification matrices between single-electrode (SE) and ROI-based responder definitions; cell color intensity reflects the count of participants in each category (darker shading indicates higher count). *k* denotes Cohen’s kappa coefficient. (**D**) Perceived difficulty ratings across four blocks for SMR (blue line) and Theta (red line) conditions; lines and shaded regions represent the mean ± SEM. (**E**) Feedback mode preference distribution; bars indicate the number of participants preferring SMR feedback (blue), Theta feedback (red), or expressing no preference (gray). (**F**) Overall satisfaction ratings (M = 8.4, Mdn = 9.0); the red dashed line marks the median. ** *p* < 0.01, *** *p* < 0.001.

Regulation Scores and Responder Classification. Among 22 participants, nine were classified as SMR responders and 13 as Theta responders (Figure 8A,B). Single-electrode and ROI-based definitions showed high agreement for SMR (κ = 0.81, z = 6.40, *p* < 0.001) and moderate agreement for Theta (κ = 0.54, z = 2.96, *p* = 0.003; Figure 8C).

Responder versus Non-Responder Neural Comparisons. The two groups showed no significant differences in pre-training resting-state global FC strength (all *ps* > 0.10), training-induced FC changes (all *ps* > 0.15), or task-state global FC (all *ps* > 0.05; Table S10, Figure S6). Channel-level power comparisons reached significance at uncorrected level for some channels but none survived FDR correction.

Perceived Difficulty. Under both SMR (χ^2^(3) = 13.42, *p* = 0.004, W = 0.20) and Theta (χ^2^(3) = 10.23, *p* = 0.017, W = 0.16) conditions, perceived difficulty progressively increased from Block 1 (Mdn ≈ 2.5–3.0) to Block 3–4 (Mdn ≈ 4.0), with no condition main effect (*p* = 0.442) or interaction (*p* = 0.695; see Figure S7 for the Condition × Block interaction trend plots; Figure 8D). Feedback helpfulness, heartbeat synchrony perception, and comprehension showed no significant between-condition differences (all *ps* > 0.13).

Mode Questionnaire and Preference. All six dimensions showed non-significant SMR–Theta differences (all *ps* > 0.15; Table S11). More participants preferred Theta over SMR across all three preference questions (Figure 8E), but binomial tests were non-significant (all *ps* > 0.21). Overall satisfaction ratings were concentrated in the high range (M = 8.36, SD = 1.36, Mdn = 9.0, range 6–10; Figure 8F).

Control Tests. Heartbeat synchrony perception (*p* = 0.861) and feedback comprehension (*p* = 0.909) showed no between-condition differences. Order effect tests were all non-significant (all *ps* > 0.29).

## 5. Discussion

Using a closed-loop BCI system based on multi-level heartbeat sound feedback, this study systematically examined the immediate effects of single-session auditory NFT on EEG rhythms, functional connectivity, and behavioral performance in moderately skilled shooters. The following three key findings emerged: first, theta feedback induced stable low-frequency power suppression in the frontal region, while SMR feedback did not produce significant sensorimotor enhancement at the group level (corresponding to H1); second, theta feedback elicited cross-frequency power coupling and functional connectivity reorganization (H2), and neural modulation effects were dissociated from behavioral performance (H3); third, NFT effects exhibited significant individual dependence (H5), and resting-state analysis provided preliminary evidence for offline effects (H4).

### 5.1. Theta-Dominant Frontal Suppression and Asymmetric SMR Regulation

The most robust finding of this study was that theta feedback induced significant and concentrated 4–7 Hz power suppression in the frontal region (cluster *p* = 0.004), directly supporting the first part of H1. Frontal midline theta is generally considered to reflect activation of the prefrontal cognitive control network, and its power reduction is closely associated with decreased cognitive monitoring load and increased task execution automatization [2,9,10]. During the shooting preparation period, FMT suppression may indicate that participants, guided by feedback, reduced their reliance on explicit cognitive resources and transitioned toward a more automatized task execution mode. This interpretation aligns with the cortical efficiency hypothesis proposed by Hatfield et al. [5]—one of the core neural markers of high-level shooting performance is selective reduction in prefrontal activity. Notably, this effect emerged within a single training session, suggesting that prefrontal theta activity possesses high short-term plasticity in response to external feedback signals, possibly related to the inherent advantage of prefrontal cortex in cognitive flexibility [70,71].

In contrast, SMR feedback did not induce significant 12–15 Hz power enhancement at the group level (*p* > 0.05), and the second part of H1 was not supported. This result is consistent with findings from the following several prior studies: Gruzelier [14] noted that the learning curve for SMR regulation is typically slower than for alpha or theta feedback, potentially requiring multiple sessions to establish stable operant conditioning. From a neurophysiological perspective, SMR enhancement depends on the local desynchronization–resynchronization cycle of sensorimotor cortex mu rhythms [8,16], and establishing this process may require participants to develop a refined strategy for inhibiting somatosensory input. For moderately skilled shooters whose motor control is still in the explicit regulation stage, the relatively high oscillatory variability of the sensorimotor system [13] may constitute a neural substrate limitation for short-term SMR regulation.

Taken together, these results support a hierarchical modulation pattern: prefrontal theta activity can be effectively regulated within a single session, whereas sensorimotor SMR regulation may require longer-term training to establish stable operant learning. This asymmetry has direct implications for NFT protocol design—in short-term intervention scenarios, targeting theta suppression may be more feasible than SMR enhancement.

### 5.2. Cross-Frequency Coupling and Functional Network Reorganization

Another important finding was that the effects of theta feedback were not confined to the target band. Among theta responders, sensorimotor SMR power was significantly reduced under theta feedback (d = −0.69), and frontal theta power change was strongly positively correlated with sensorimotor SMR power change (r = 0.67, *p* < 0.001), with the correlation further strengthening in responders (r = 0.82). This result partially supports H2, indicating that target-band regulation indeed influenced non-target band activity through inter-cortical functional coupling.

From a mechanistic perspective, this cross-frequency coupling may reflect the functional coupling relationship between frontal and sensorimotor networks. Prior research has demonstrated that during action execution, low-frequency oscillatory activity in prefrontal regions shows significantly enhanced functional connectivity with the sensorimotor network [72]. Frontal midline theta oscillations are considered a key neural channel for implementing cognitive control that can modulate the activity states of multiple downstream networks, including the motor system [71]. Systematic reduction in prefrontal activity may “drag” sensorimotor cortex activity levels through this pathway, manifesting as global cortical power downregulation. Notably, this pattern is inconsistent with the classical hypofrontality hypothesis [11,12], which predicts prefrontal suppression accompanied by sensorimotor enhancement. One plausible explanation is that during the early stages of single-session training, individuals have not yet established differentiated frequency-band regulation strategies, and the rapid prefrontal adjustment produces a non-specific accompanying effect on the sensorimotor area through functional coupling; with multiple training sessions, stabilization of prefrontal regulation may progressively release the sensorimotor area’s autonomous regulation capacity, allowing the hypofrontality pattern to eventually emerge. Empirical studies have also shown that the hypofrontality phenomenon exhibits degree-dependent differences across different exercise intensities and task automatization levels [11], and the degree of prefrontal theta suppression is closely related to the progression from executive control toward automatized processing [67], which is consistent with the theoretical predictions of the hierarchical modulation pattern described above.

Functional connectivity analysis provided complementary network-level evidence. SMR feedback induced a significant whole-brain reduction in theta-band connectivity in the full sample (cluster statistic = −128.60, *p* = 0.034), with the effect more pronounced in theta responders (*p* = 0.024), suggesting that connectivity reorganization and power regulation share a similar individual-difference structure. Particularly noteworthy is that theta non-responders, despite failing to effectively regulate theta power, exhibited significant SMR-band connectivity reductions (*p* = 0.003), suggesting that functional network reorganization may not entirely depend on successful target-band power regulation but may partly reflect broader perturbation effects of auditory feedback on the neural system. This finding extends the existing understanding that single-band feedback can induce multi-band responses [15,17], further revealing that power coupling and connectivity reorganization may follow different regulatory logics: the former depends more on individual target regulation ability, while the latter may have greater universality.

### 5.3. Dual-Task Interference and Neural–Behavioral Dissociation

A feature of the task-embedded auditory NFT paradigm that has been less thoroughly examined in the existing literature is that the feedback channel is not a neutral information delivery conduit but may itself constitute a parallel secondary task. To our knowledge, prior task-embedded auditory NFT studies in the precision motor domain [18,21,22] focus primarily on pre-training versus post-training behavioral comparisons and do not appear to separately report behavioral performance during the feedback period itself. By introducing a no-sound baseline as a control for the feedback conditions within the same participants and the same session, this study provides relatively direct evidence for examining behavioral changes during the feedback period. Consistent with the predicted direction: shooting ring values did not improve under either feedback condition, aiming time was significantly prolonged (F(2, 42) = 7.020, *p* = 0.002), and theta feedback non-responders also exhibited significantly reduced horizontal deviation (d = −1.75) but accompanied by longer aiming time. Such within-session neural–behavioral dissociation echoes earlier reports in the motor neurofeedback literature [73]. This pattern is consistent with dual-task theory predictions [69,70]: when limited central resources must be allocated between aiming/postural control and feedback monitoring, speed–accuracy trade-offs may emerge [74,75].

Despite significant neural modulation effects, shooting ring values showed no significant change at the group level or in any subgroup (*p* = 0.160), consistent with H3 and supporting the neural–behavioral dissociation perspective. This dissociation has been reported in the motor neurofeedback literature: Ring et al. [73] similarly found in a golf putting study that NFT successfully modulated target-band cortical activity but did not produce behavioral improvements beyond those of the control group.

The neural–behavioral dissociation in this study can be attributed to at least two mechanisms. First, the competitive effect of dual-task cognitive load: the experimental context inherently created a dual-task structure, with participants simultaneously performing shooting while monitoring auditory feedback and attempting neural regulation [23,24]. For moderately skilled shooters whose motor patterns are not yet automatized, cognitive resources are already largely consumed by postural control and visual aiming, and additional feedback monitoring may further increase endogenous cognitive load [26,27], thereby offsetting the potential behavioral benefits of neural regulation. Second, the limitation of single-session training duration: behavioral improvement may require neural regulation patterns to be internalized as stable procedural memory through multiple training sessions [14,34,35], and a single 90 min session is insufficient to complete the entire process from neural plasticity to behavioral transfer.

Notably, behavioral data revealed a “behavioral compensation pathway” distinct from the neural regulation pathway: aiming time was significantly prolonged under both feedback conditions (*p* = 0.002), and theta feedback non-responders showed significantly reduced horizontal deviation (d = −1.75). This pattern suggests that some participants may have strategically prolonged aiming time to improve spatial accuracy rather than relying on neural state improvements. This behavioral compensation mechanism has been described in the motor learning literature as a speed–accuracy trade-off [74,75], and its occurrence further supports the multi-pathway relationship between neural regulation and behavioral improvement, rather than simple linear transmission.

The auditory channel can act on the prefrontal cognitive control network at the neural level, and heartbeat sounds as a feedback carrier performed well in terms of subjective acceptance. However, this auditory channel, while embedded in the motor task, also introduces measurable dual-task costs at the behavioral execution level. The absence of such costs in prior “task + feedback” designs may be attributable, at least in part, to the fact that those studies, to our knowledge, did not separately report behavioral performance during the feedback period itself [18,21,22]. This finding carries practical implications as follows: in single-session training designs, behavioral gains observed after training should not be directly equated with the neural processes occurring during the feedback period; improving the design of task-embedded feedback (e.g., reducing its decoding burden, sparsifying feedback presentation frequency, or delivering some feedback offline during inter-trial intervals) may be equally important as enhancing the quality of the neural signal itself.

### 5.4. Resting-State Offline Effects

Eyes-closed resting-state analysis showed that post-training Fz theta power (d = −0.52) and Cz SMR power (d = −0.46) were both significantly reduced compared to pre-training, providing preliminary support for H4 and suggesting that single-session NFT may produce lasting effects on spontaneous brain activity. However, interpretation of these results requires careful consideration of confounding factors.

First, the direction of SMR power reduction was opposite to the training goal (enhancement), more likely reflecting neural fatigue from the prolonged experiment (approximately 90 min) rather than training-induced plasticity—sustained cognitive and motor load may cause an overall decline in cortical arousal [76,77]. This interpretation is supported by the absence of similar changes under the eyes-open condition, suggesting that the eyes-closed power reduction more likely reflects short-term adaptive adjustment of resting-state networks rather than lasting neuroplastic remodeling. Goodman et al. [78] further confirmed that sustained cognitive load can induce measurable multidimensional neurophysiological changes, supporting the fatigue interpretation. Second, resting-state functional connectivity did not reach significance at the cluster level; only the Fz–Cz theta-band connection under the eyes-open condition showed a significant reduction (d = −0.54), consistent in direction with the task-state connectivity reorganization trend, but its functional significance requires verification in multi-session paradigms.

Overall, the resting-state results provide preliminary indications of offline effects, but due to the absence of an active control condition (e.g., sham feedback), the observed changes cannot be entirely attributed to NFT per se; fatigue, habituation, and time effects cannot be excluded as confounds.

### 5.5. Individual Differences and BCI Illiteracy

The SMR responder rate was 40.9% (9/22) and the theta responder rate was 59.1% (13/22), with non-responder proportions at the higher end of the 15–30% range reported in the literature [30,31], supporting H5. The elevated non-response rate may relate to the single-session design: limited training opportunities may be insufficient for participants to develop precise discrimination of target internal states and consequently fail to develop stable self-maintenance strategies [79,80]. Furthermore, NFT learning is more akin to a skill acquisition process than simple conditioning [81], and some participants may be “late learners” requiring additional training sessions.

Responders and non-responders exhibited differentiated regulation pathways at both neural and behavioral levels. At the neural level, theta responders drove the majority of the whole-sample functional connectivity effects (see Section 4.2), while non-responders exhibited significant SMR-band connectivity reorganization despite failing to regulate target-band power. At the behavioral level, non-responders showed stronger behavioral compensation under theta feedback (horizontal deviation d = −1.75), suggesting that when the neural regulation pathway is obstructed, individuals may shift toward behavioral strategy adjustment to meet task demands. This differentiation between “neural regulation pathway” and “behavioral compensation pathway” enriches our understanding of BCI illiteracy—non-responders are not completely “non-responsive” but may employ alternative adaptation strategies.

However, it should be noted that responders and non-responders did not differ significantly in resting-state baseline characteristics (*ps* > 0.100), and channel-level power comparisons were all non-significant after FDR correction, suggesting that group differences may stem more from differences in online learning ability than from static neural substrate characteristics.

### 5.6. Design Considerations for Heartbeat Sound Feedback

The use of heartbeat sounds as the feedback carrier in place of conventional pure tones merits separate discussion. Subjective evaluation data showed high overall system satisfaction (M = 8.36/10), and the two feedback modes showed no significant differences in heartbeat synchrony perception or feedback comprehension (*ps* > 0.130), indicating good subjective acceptance of the heartbeat sound feedback. The progressive increase in perceived difficulty across blocks (SMR: *p* = 0.004; Theta: *p* = 0.017) indirectly reflects the effectiveness of the dynamic threshold mechanism—the system successfully escalated training challenge based on individual performance. Gong et al. [55] found that perceived difficulty differed significantly across feedback modes, highlighting the importance of feedback design for user experience. Gadea et al. [82] similarly observed that a single SMR–NFT session was sufficient to significantly reduce participants’ subjective anxiety, demonstrating that even brief interventions can affect immediate psychological experience through well-designed feedback systems.

From a theoretical perspective, heartbeat sounds—as interoception-related physiological rhythm signals—may offer advantages in the following two respects: first, higher biological familiarity may reduce the cognitive intrusiveness of feedback signals, relieving participants from allocating additional attentional resources to decode feedback meaning [32,33,40]; second, the dual-channel rate–intensity encoding (see Table 1) provides a richer state information gradient than single pure tones. However, as this study did not include a pure-tone feedback control group, these advantages remain theoretical speculation and cannot be verified at the experimental level. Future studies could quantify the specific benefits of interoception-related feedback in terms of cognitive load and regulation efficiency through direct comparison of heartbeat sound and pure-tone feedback.

### 5.7. Limitations and Future Directions

This study has the following limitations. First, the single-session design may be insufficient to induce stable SMR regulation effects and behavioral transfer; future research should employ multi-session paradigms (e.g., 10–20 sessions) to systematically assess learning curves, long-term plasticity, and the temporal course of behavioral gains. Second, the sample size (*N* = 22) provided adequate power for large effect sizes (1 − β = 0.94, d = 0.80) but limited sensitivity for medium effects (1 − β = 0.59, d = 0.50), with subgroup comparisons particularly underpowered; future studies should expand to 40 or more participants. Third, the fixed-order placement of the no-sound baseline condition, while avoiding carryover contamination, may introduce practice or fatigue confounds; future designs could incorporate a separate baseline testing day or randomize the baseline condition. Fourth, the absence of a sham feedback control group precludes attributing observed neural changes entirely to operant conditioning rather than non-specific auditory stimulation or placebo effects. Including a fourth condition within a single session would extend the total duration beyond the 90–120 min reasonable range, and pilot testing indicated that data quality deteriorated markedly beyond this boundary. It should be noted that theta feedback and SMR feedback exhibited clearly different neural response patterns in this study (the former induced significant frontal suppression while the latter showed no significant group-level enhancement), and theta responders displayed cross-frequency coupling correlated with regulation success (r = 0.67, *p* < 0.001). These frequency-specific and individual-difference characteristics are not fully consistent with predictions of general auditory stimulation effects, although this alternative explanation cannot be entirely ruled out in the absence of sham control. Future studies could incorporate an acoustically matched sham feedback condition to further disentangle the contributions of feedback contingency and non-specific auditory stimulation. Fifth, the sex ratio was substantially unbalanced (20 males, two females), primarily due to the objective structural characteristics of the recruitment pool: participants were drawn from the university’s shooting elective course, which was predominantly enrolled by male students, so the sample sex distribution reflects the characteristics of the recruitment population rather than active screening. However, this imbalance does limit the generalizability of the conclusions to female populations. Future studies could consider actively balancing the sex ratio during recruitment, or incorporating sex as a stratification factor in the analysis to test the generalizability of the present findings. Sixth, as all participants were moderately skilled shooters, extrapolation to higher skill levels requires caution.

Despite these limitations, the core findings were consistent across multiple analytical strategies including whole-brain cluster tests, ROI validation, functional connectivity analysis, and responder definition robustness checks, indicating good statistical reliability. Future work should verify and extend these findings within frameworks incorporating multi-session training, larger samples, sham feedback controls, and cross-skill-level comparisons.

## 6. Conclusions

This study designed and implemented a closed-loop BCI system based on multi-level heartbeat sound feedback, systematically examining the immediate effects of single-session auditory NFT on EEG rhythms, functional connectivity, and behavioral performance in moderately skilled shooters at the whole-brain scale. Theta feedback stably induced significant frontal 4–7 Hz power suppression during the pre-shot preparation period, concentrated in the −2.2 s to −1.6 s time window, suggesting that the prefrontal cognitive control network possesses high short-term plasticity in response to external auditory feedback. In contrast, SMR feedback did not produce significant 12–15 Hz power enhancement at the group level, indicating that operant regulation of sensorimotor rhythms may require longer-term training to be stably established.

The effects of theta feedback were not limited to the target band—sensorimotor SMR power was concurrently reduced in theta responders, and frontal theta change was significantly positively correlated with sensorimotor SMR change (r = 0.67), revealing a cross-frequency functional coupling relationship between prefrontal and sensorimotor networks. Functional connectivity analysis further demonstrated that NFT not only modulated local band power but also triggered significant reorganization of theta-band whole-brain connectivity networks, and that power regulation and connectivity reorganization may follow different individual-difference logics.

Despite clear neural modulation effects, shooting ring values did not significantly improve at the group or subgroup levels, supporting the neural–behavioral dissociation prediction—under the dual constraints of dual-task cognitive load and single-session training duration, neural state optimization precedes behavioral performance improvement. Behavioral data simultaneously revealed a “behavioral compensation pathway” based on prolonged aiming time, suggesting a multi-pathway rather than single linear relationship between neural regulation and behavioral improvement. Additionally, approximately 40–60% of participants were classified as non-responders, who exhibited differentiated adaptation pathways in both neural regulation patterns and behavioral strategies, underscoring the methodological necessity of distinguishing individual response patterns when evaluating NFT effects.

Overall, this study demonstrates that single-session auditory NFT can serve as an effective neural state modulation tool, preferentially acting on prefrontal cognitive control processes and influencing broader cortical networks through cross-frequency coupling. The multi-level heartbeat sound feedback design performed well in terms of subjective acceptance and system usability, providing both a system design reference and empirical evidence for the application of auditory NFT in precision motor skill training. Future research should further verify long-term plasticity effects and their transfer to the behavioral level within frameworks incorporating multi-session training, larger samples, and sham feedback controls.

## Figures and Tables

**Table 1:** Acoustic parameters of the five-level heartbeat sound feedback.

Level	State	Volume Coefficient	Playback Rate	Heartbeat Pattern	Fade-In/Fade-Out Duration
1	Optimal (deep focus)	0.20×	0.55×	Single gentle heartbeat	150/150 ms
2	Good	0.35×	0.70×	Single heartbeat	100/100 ms
3	Baseline (normal)	0.60×	0.85×	Normal-rhythm heartbeat	60/60 ms
4	Deviation	0.90×/0.60×	1.10×	Double heartbeat (80 ms interval)	40/40 ms
5	Extreme (needs adjustment)	1.00/0.75/0.50×	1.30×	Triple heartbeat (60 ms interval)	30/30 ms

Note. Volume coefficients represent the ratio relative to the original heartbeat audio amplitude; playback rates represent the multiplier relative to the original audio sampling rate. In Levels 4 and 5, the volume of successive heartbeats follows a descending sequence—loudest on the first beat and progressively attenuating—to simulate the perceptual characteristics of a racing heartbeat under stress.

## Data Availability

The data presented in this study are available on request from the corresponding author. The data are not publicly available due to privacy concerns related to study participants.

## Supplementary Materials

The following supporting information can be downloaded at https://www.mdpi.com/article/10.3390/s26103187/s1, Table S1: Post hoc power analysis reference table; Table S2: Responder classification agreement; Table S3: Outlier sensitivity analysis; Table S4: Cluster-based permutation test detailed parameters; Table S5: ROI paired *t*-tests and cross-frequency correlation analyses; Table S6: Task-state functional connectivity statistics; Table S7: Complete statistical results for all seven behavioral indicators; Table S8: Resting-state power statistics; Table S9: Resting-state functional connectivity and graph-theoretic statistics; Table S10: Multi-dimensional comparison between responders and non-responders; Table S11: Complete subjective evaluation questionnaire statistics; Figure S1: Data quality diagnostic panel; Figure S2: Outlier sensitivity analysis visualization; Figure S3: Task-state functional connectivity detail plots; Figure S4: Supplementary behavioral results; Figure S5: Resting-state eyes-open condition detail plots; Figure S6: Per-participant regulation score distributions; Figure S7: MiniQ Condition × Block interaction trend plots. Table S12: Impact of window overlap on trial success rate—robustness analysis (per-participant, per-block success rate comparison between overlapping “two-of-four” and non-overlapping “two-of-three” schemes with paired test results).

## Abbreviations

The following abbreviations are used in this manuscript:

BCI	Brain–Computer Interface
NFT	Neurofeedback Training
EEG	Electroencephalography
SMR	Sensorimotor Rhythm
FMT	Frontal Midline Theta
FIR	Finite Impulse Response
IIR	Infinite Impulse Response
dwPLI	Debiased Weighted Phase Lag Index
SCD	Scalp Current Density
ICA	Independent Component Analysis
ROI	Region of Interest
RM-ANOVA	Repeated-Measure Analysis of Variance
FDR	False Discovery Rate
FFT	Fast Fourier Transform
EC	Eyes Closed
EO	Eyes Open
FC	Functional Connectivity

## References

[1] Hatfield B.D. (2018). Brain Dynamics and Motor Behavior: A Case for Efficiency and Refinement for Superior Performance. Kinesiol. Rev..

[2] Hatfield B.D., Haufler A.J., Hung T.-M., Spalding T.W. (2004). Electroencephalographic Studies of Skilled Psychomotor Performance. J. Clin. Neurophysiol..

[3] Hatfield B.D., Hillman C.H., Singer R.N., Hausenblas H.A., Janelle C.M. (2001). The Psychophysiology of Sport: A Mechanistic Understanding of the Psychology of Superior Performance. Handbook of Sport Psychology.

[4] Li L., Smith D.M. (2021). Neural Efficiency in Athletes: A Systematic Review. Front. Behav. Neurosci..

[5] Haufler A.J., Spalding T.W., Santa Maria D.L. (2000). Neuro-Cognitive Activity during a Self-Paced Visuospatial Task: Comparative EEG Profiles in Marksmen and Novice Shooters. Biol. Psychol..

[6] Del Percio C., Babiloni C., Bertollo M., Marzano N., Iacoboni M., Infarinato F., Lizio R., Stocchi M., Robazza C., Cibelli G. (2009). Visuo-Attentional and Sensorimotor Alpha Rhythms Are Related to Visuo-Motor Performance in Athletes. Hum. Brain Mapp..

[7] Doppelmayr M., Finkenzeller T., Sauseng P. (2008). Frontal Midline Theta in the Pre-Shot Phase of Rifle Shooting: Differences between Experts and Novices. Neuropsychologia.

[8] Cheng M.-Y., Wang K.-P., Hung C.-L., Tu Y.-L., Huang C.-J., Koester D., Schack T., Hung T.-M. (2017). Higher Power of Sensorimotor Rhythm Is Associated with Better Performance in Skilled Air-Pistol Shooters. Psychol. Sport Exerc..

[9] Deeny S.P., Haufler A.J., Saffer M., Hatfield B.D. (2009). Electroencephalographic Coherence during Visuomotor Performance: A Comparison of Cortico-Cortical Communication in Experts and Novices. J. Mot. Behav..

[10] Deeny S.P., Hillman C.H., Janelle C.M., Hatfield B.D. (2003). Cortico-Cortical Communication and Superior Performance in Skilled Marksmen: An EEG Coherence Analysis. J. Sport Exerc. Psychol..

[11] Filho E., Dobersek U., Husselman T.-A. (2021). The Role of Neural Efficiency, Transient Hypofrontality and Neural Proficiency in Optimal Performance in Self-Paced Sports: A Meta-Analytic Review. Exp. Brain Res..

[12] Raman D., Filho E. (2024). The Relationship between T7-Fz Alpha Coherence and Peak Performance in Self-Paced Sports: A Meta-Analytical Review. Exp. Brain Res..

[13] Neubauer A.C., Fink A. (2009). Intelligence and Neural Efficiency: Measures of Brain Activation versus Measures of Functional Connectivity in the Brain. Intelligence.

[14] Gruzelier J.H. (2014). EEG-Neurofeedback for Optimising Performance. I: A Review of Cognitive and Affective Outcome in Healthy Participants. Neurosci. Biobehav. Rev..

[15] Egner T., Gruzelier J.H. (2004). EEG Biofeedback of Low Beta Band Components: Frequency-Specific Effects on Variables of Attention and Event-Related Brain Potentials. Clin. Neurophysiol..

[16] Gruzelier J.H. (2014). EEG-Neurofeedback for Optimising Performance. II: Creativity, the Performing Arts and Ecological Validity. Neurosci. Biobehav. Rev..

[17] Enriquez-Geppert S., Huster R.J., Herrmann C.S. (2017). EEG-Neurofeedback as a Tool to Modulate Cognition and Behavior: A Review Tutorial. Front. Hum. Neurosci..

[18] Cheng M.-Y., Huang C.-J., Chang Y.-K., Koester D., Schack T., Hung T.-M. (2015). Sensorimotor Rhythm Neurofeedback Enhances Golf Putting Performance. J. Sport Exerc. Psychol..

[19] Rostami R., Sadeghi H., Karami K.A., Abadi M.N., Salamati P. (2012). The Effects of Neurofeedback on the Improvement of Rifle Shooters’ Performance. J. Neurother..

[20] Bakhtafrooz S., Kavyani M., Farsi A., Alboghebeish S. (2025). The Effect of Infra Low Frequency–Neurofeedback Training on Pistol Shooting Performance and Attention in Semi-Skilled Players. Front. Hum. Neurosci..

[21] Toolis T., Cooke A., Laaksonen M.S., McGawley K. (2023). Effects of Neurofeedback Training on Frontal Midline Theta Power, Shooting Performance, and Attentional Focus with Experienced Biathletes. J. Clin. Sport Psychol..

[22] Wu J., Chueh T., Yu C., Wang K., Kao S., Gentili R.J., Hatfield B.D., Hung T. (2024). Effect of a Single Session of Sensorimotor Rhythm Neurofeedback Training on the Putting Performance of Professional Golfers. Scand. J. Med. Sci. Sports.

[23] Pashler H. (1994). The Ubiquitous PRP Effect. Psychol. Bull..

[24] Wickens C.D. (2002). Multiple Resources and Performance Prediction. Theor. Issues Ergon. Sci..

[25] Jeunet C., Lotte F., Batail J.-M., Philip P., Micoulaud Franchi J.-A. (2018). Using Recent BCI Literature to Deepen Our Understanding of Clinical Neurofeedback: A Short Review. Neuroscience.

[26] Sweller J., Van Merriënboer J.J.G., Paas F. (2019). Cognitive Architecture and Instructional Design: 20 Years Later. Educ. Psychol. Rev..

[27] Sweller J. (1988). Cognitive Load during Problem Solving: Effects on Learning. Cogn. Sci..

[28] Sidhu A., Cooke A. (2021). Electroencephalographic Neurofeedback Training Can Decrease Conscious Motor Control and Increase Single and Dual-Task Psychomotor Performance. Exp. Brain Res..

[29] Orendacova M., Kvasnak E. (2025). What Can Neurofeedback and Transcranial Alternating Current Stimulation Reveal about Cross-Frequency Coupling?. Front. Neurosci..

[30] Allison B.Z., Neuper C., Tan D.S., Nijholt A. (2010). Could Anyone Use a BCI?. Brain–Computer Interfaces.

[31] Vidaurre C., Blankertz B. (2010). Towards a Cure for BCI Illiteracy. Brain Topogr..

[32] Dobrushina O., Tamim Y., Wald I.Y., Maimon A., Amedi A. (2024). Interoceptive Training with Real-Time Haptic versus Visual Heartbeat Feedback. Psychophysiology.

[33] Greenwood B.M., Garfinkel S.N. (2025). Interoceptive Mechanisms and Emotional Processing. Annu. Rev. Psychol..

[34] Robertson E.M., Pascual-Leone A., Miall R.C. (2004). Current Concepts in Procedural Consolidation. Nat. Rev. Neurosci..

[35] Dayan E., Cohen L.G. (2011). Neuroplasticity Subserving Motor Skill Learning. Neuron.

[36] Huang D., Wang Y., Fan L., Yu Y., Zhao Z., Zeng P., Wang K., Li N., Shen H. (2024). Decoding Subject-Driven Cognitive States from EEG Signals for Cognitive Brain–Computer Interface. Brain Sci..

[37] Yang B., Rong F., Xie Y., Li D., Zhang J., Li F., Shi G., Gao X. (2025). A Multi-Day and High-Quality EEG Dataset for Motor Imagery Brain-Computer Interface. Sci. Data.

[38] Widmann A., Schröger E., Maess B. (2015). Digital Filter Design for Electrophysiological Data—A Practical Approach. J. Neurosci. Methods.

[39] Donoghue T., Haller M., Peterson E.J., Varma P., Sebastian P., Gao R., Noto T., Lara A.H., Wallis J.D., Knight R.T. (2020). Parameterizing Neural Power Spectra into Periodic and Aperiodic Components. Nat. Neurosci..

[40] Murphy J., Brewer R., Plans D., Khalsa S.S., Catmur C. (2020). Testing the Independence of Self-Reported Interoceptive Accuracy and Attention. Q. J. Exp. Psychol..

[41] Fleury M., Lioi G., Barillot C., Lécuyer A. (2020). A Survey on the Use of Haptic Feedback for Brain-Computer Interfaces and Neurofeedback. Front. Neurosci..

[42] Omejc N., Rojc B., Battaglini P.P., Marusic U. (2018). Review of the Therapeutic Neurofeedback Method Using Electroencephalography: EEG Neurofeedback. Bosn. J. Basic Med. Sci..

[43] Takabatake K., Kunii N., Nakatomi H., Shimada S., Yanai K., Takasago M., Saito N. (2021). Musical Auditory Alpha Wave Neurofeedback: Validation and Cognitive Perspectives. Appl. Psychophysiol. Biofeedback.

[44] Cheng M.-Y., Yu C.-L., An X., Wang L., Tsai C.-L., Qi F., Wang K.-P. (2024). Evaluating EEG Neurofeedback in Sport Psychology: A Systematic Review of RCT Studies for Insights into Mechanisms and Performance Improvement. Front. Psychol..

[45] Yu C.-L., Cheng M.-Y., An X., Chueh T.-Y., Wu J.-H., Wang K.-P., Hung T.-M. (2025). The Effect of EEG Neurofeedback Training on Sport Performance: A Systematic Review and Meta-Analysis. Scand. J. Med. Sci. Sports.

[46] Xiang M.-Q., Hou X.-H., Liao B.-G., Liao J.-W., Hu M. (2018). The Effect of Neurofeedback Training for Sport Performance in Athletes: A Meta-Analysis. Psychol. Sport Exerc..

[47] Weber L.A., Ethofer T., Ehlis A.-C. (2020). Predictors of Neurofeedback Training Outcome: A Systematic Review. NeuroImage Clin..

[48] Bale J.H., Wilkinson M. (2023). Validity and Reliability of an Opto-Electric Training System in Elite and National Level ISSF Air Rifle Shooters. Sports Eng..

[49] Mon-López D., De La Rubia A., García-Aliaga A., Acebes-Sánchez J., Refoyo Roman I., Lorenzo Calvo J. (2022). Optoelectronic Analysis of Technical Factors and Performance of Elite-Level Air Pistol Shooting. PLoS ONE.

[50] Michalska J., Zajac R., Szydlo K., Gerasimuk D., Slomka K.J., Juras G. (2022). Biathletes Present Repeating Patterns of Postural Control to Maintain Their Balance While Shooting. PLoS ONE.

[51] Mononen K., Konttinen N., Viitasalo J., Era P. (2007). Relationships between Postural Balance, Rifle Stability and Shooting Accuracy among Novice Rifle Shooters. Scand. J. Med. Sci. Sports.

[52] Spancken S., Steingrebe H., Stein T. (2021). Factors That Influence Performance in Olympic Air-Rifle and Small-Bore Shooting: A Systematic Review. PLoS ONE.

[53] Ros T., Enriquez-Geppert S., Zotev V., Young K.D., Wood G., Whitfield-Gabrieli S., Wan F., Vuilleumier P., Vialatte F., Van De Ville D. (2020). Consensus on the Reporting and Experimental Design of Clinical and Cognitive-Behavioural Neurofeedback Studies (CRED-Nf Checklist). Brain.

[54] Su K.-H., Hsueh J.-J., Chen T., Shaw F.-Z. (2021). Validation of Eyes-Closed Resting Alpha Amplitude Predicting Neurofeedback Learning of Upregulation Alpha Activity. Sci. Rep..

[55] Gong A., Nan W., Yin E., Jiang C., Fu Y. (2020). Efficacy, Trainability, and Neuroplasticity of SMR vs. Alpha Rhythm Shooting Performance Neurofeedback Training. Front. Hum. Neurosci..

[56] Delorme A., Makeig S. (2004). EEGLAB: An Open Source Toolbox for Analysis of Single-Trial EEG Dynamics Including Independent Component Analysis. J. Neurosci. Methods.

[57] Delorme A., Sejnowski T., Makeig S. (2007). Enhanced Detection of Artifacts in EEG Data Using Higher-Order Statistics and Independent Component Analysis. NeuroImage.

[58] Pion-Tonachini L., Kreutz-Delgado K., Makeig S. (2019). ICLabel: An Automated Electroencephalographic Independent Component Classifier, Dataset, and Website. NeuroImage.

[59] Chaumon M., Bishop D.V.M., Busch N.A. (2015). A Practical Guide to the Selection of Independent Components of the Electroencephalogram for Artifact Correction. J. Neurosci. Methods.

[60] Yao D., Qin Y., Hu S., Dong L., Bringas Vega M.L., Valdés Sosa P.A. (2019). Which Reference Should We Use for EEG and ERP Practice?. Brain Topogr..

[61] Oostenveld R., Fries P., Maris E., Schoffelen J.-M. (2011). FieldTrip: Open Source Software for Advanced Analysis of MEG, EEG, and Invasive Electrophysiological Data. Comput. Intell. Neurosci..

[62] Cohen M.X. (2019). A Better Way to Define and Describe Morlet Wavelets for Time-Frequency Analysis. NeuroImage.

[63] Vinck M., Oostenveld R., Van Wingerden M., Battaglia F., Pennartz C.M.A. (2011). An Improved Index of Phase-Synchronization for Electrophysiological Data in the Presence of Volume-Conduction, Noise and Sample-Size Bias. NeuroImage.

[64] Rubinov M., Sporns O. (2010). Complex Network Measures of Brain Connectivity: Uses and Interpretations. NeuroImage.

[65] Cohen J. (1988). Statistical Power Analysis for the Behavioral Sciences.

[66] Maris E., Oostenveld R. (2007). Nonparametric Statistical Testing of EEG- and MEG-Data. J. Neurosci. Methods.

[67] Sassenhagen J., Draschkow D. (2019). Cluster-Based Permutation Tests of MEG/EEG Data Do Not Establish Significance of Effect Latency or Location. Psychophysiology.

[68] Nichols T.E., Holmes A.P. (2002). Nonparametric Permutation Tests for Functional Neuroimaging: A Primer with Examples. Hum. Brain Mapp..

[69] Steegen S., Tuerlinckx F., Gelman A., Vanpaemel W. (2016). Increasing Transparency through a Multiverse Analysis. Perspect. Psychol. Sci..

[70] Chikhi S., Matton N., Sanna M., Blanchet S. (2024). Effects of One Session of Theta or High Alpha Neurofeedback on EEG Activity and Working Memory. Cogn. Affect. Behav. Neurosci..

[71] Cavanagh J.F., Frank M.J. (2014). Frontal Theta as a Mechanism for Cognitive Control. Trends Cogn. Sci..

[72] Babiloni C., Del Percio C., Lopez S., Di Gennaro G., Quarato P.P., Pavone L., Morace R., Soricelli A., Noce G., Esposito V. (2017). Frontal Functional Connectivity of Electrocorticographic Delta and Theta Rhythms during Action Execution versus Action Observation in Humans. Front. Behav. Neurosci..

[73] Ring C., Cooke A., Kavussanu M., McIntyre D., Masters R. (2015). Investigating the Efficacy of Neurofeedback Training for Expediting Expertise and Excellence in Sport. Psychol. Sport Exerc..

[74] Fitts P.M. (1954). The Information Capacity of the Human Motor System in Controlling the Amplitude of Movement. J. Exp. Psychol..

[75] Schmidt R.A., Zelaznik H., Hawkins B., Frank J.S., Quinn J.T. (1979). Motor-Output Variability: A Theory for the Accuracy of Rapid Motor Acts. Psychol. Rev..

[76] Li G., Huang S., Xu W., Jiao W., Jiang Y., Gao Z., Zhang J. (2020). The Impact of Mental Fatigue on Brain Activity: A Comparative Study Both in Resting State and Task State Using EEG. BMC Neurosci..

[77] Barwick F., Arnett P., Slobounov S. (2012). EEG Correlates of Fatigue during Administration of a Neuropsychological Test Battery. Clin. Neurophysiol..

[78] Goodman S.P.J., Collins B., Shorter K., Moreland A.T., Papic C., Hamlin A.S., Kassman B., Marino F.E. (2025). Approaches to Inducing Mental Fatigue: A Systematic Review and Meta-Analysis of (Neuro)Physiologic Indices. Behav. Res. Methods.

[79] Autenrieth M., Kober S.E., Neuper C., Wood G. (2020). How Much Do Strategy Reports Tell about the Outcomes of Neurofeedback Training? A Study on the Voluntary Up-Regulation of the Sensorimotor Rhythm. Front. Hum. Neurosci..

[80] Gaume A., Vialatte A., Mora-Sánchez A., Ramdani C., Vialatte F.B. (2016). A Psychoengineering Paradigm for the Neurocognitive Mechanisms of Biofeedback and Neurofeedback. Neurosci. Biobehav. Rev..

[81] Veilahti A.V.P., Kovarskis L., Cowley B.U. (2021). Neurofeedback Learning Is Skill Acquisition but Does Not Guarantee Treatment Benefit: Continuous-Time Analysis of Learning-Curves from a Clinical Trial for ADHD. Front. Hum. Neurosci..

[82] Gadea M., Aliño M., Hidalgo V., Espert R., Salvador A. (2020). Effects of a Single Session of SMR Neurofeedback Training on Anxiety and Cortisol Levels. Neurophysiol. Clin..

