# Supplementary figures and images for: Design and Experimental Investigation of a Multi-Level Heartbeat Sound Feedback-Based Neurofeedback System: Neural Mechanisms
Source: Sensors (Basel). 2026 May 18;26(10):3187. doi: 10.3390/s26103187 (PMC13210765; doi:10.3390/s26103187)

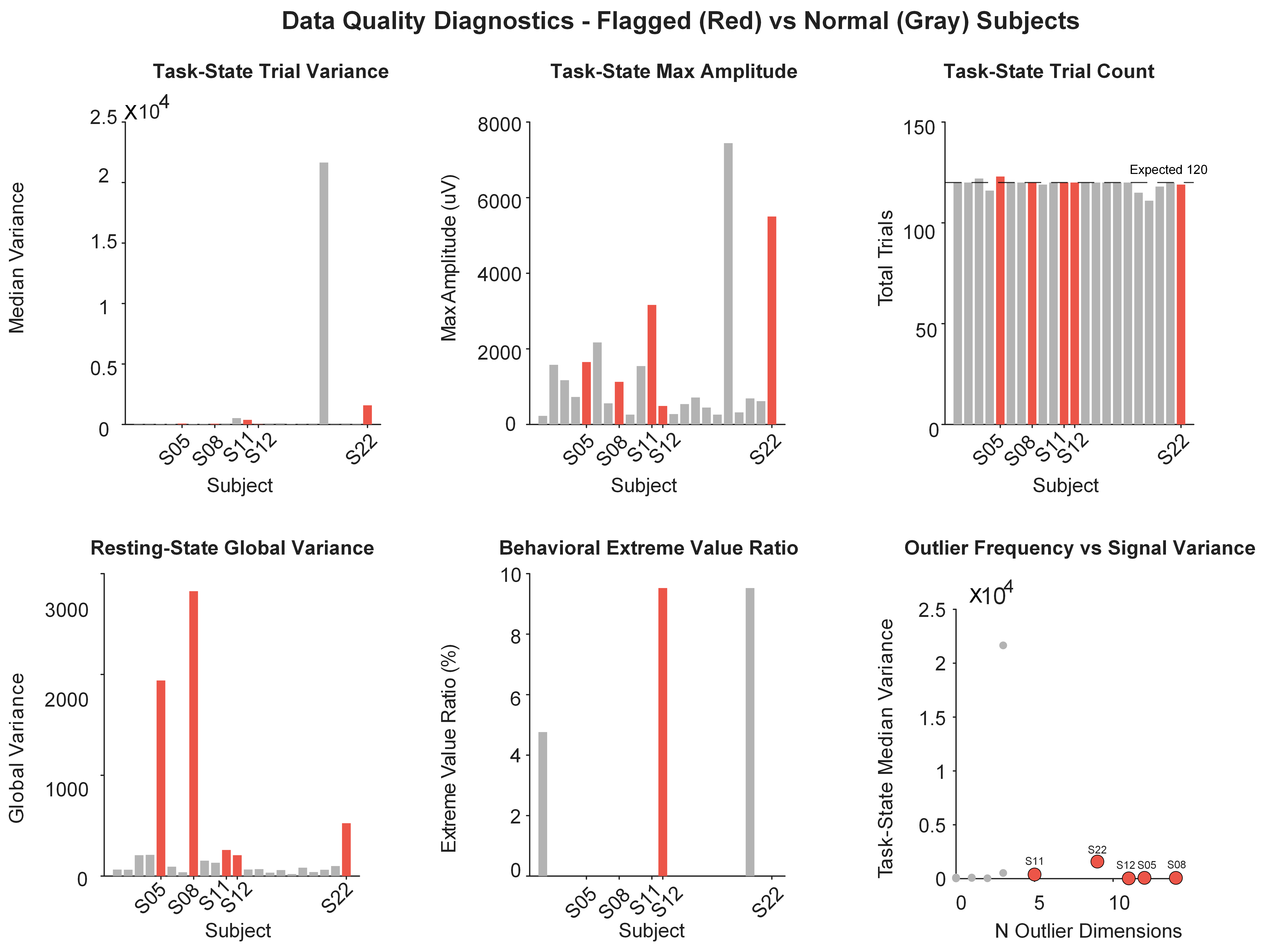

Supplement: Supplementary file 1 [file sensors-26-03187-s001.zip › Supplementary/Figure_S1_DataQuality.png]

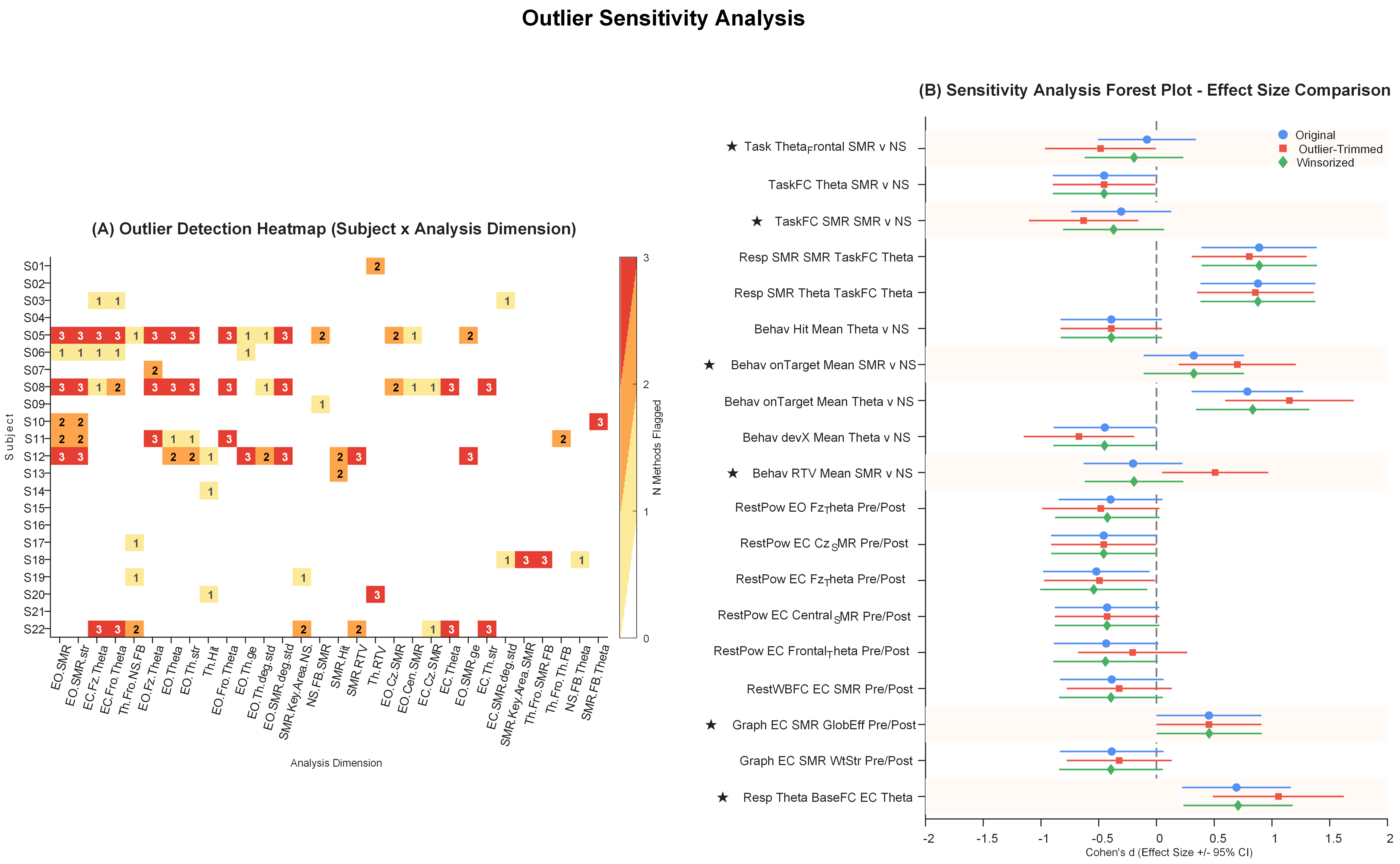

Supplement: Supplementary file 1 [file sensors-26-03187-s001.zip › Supplementary/Figure_S2_Outlier.png]

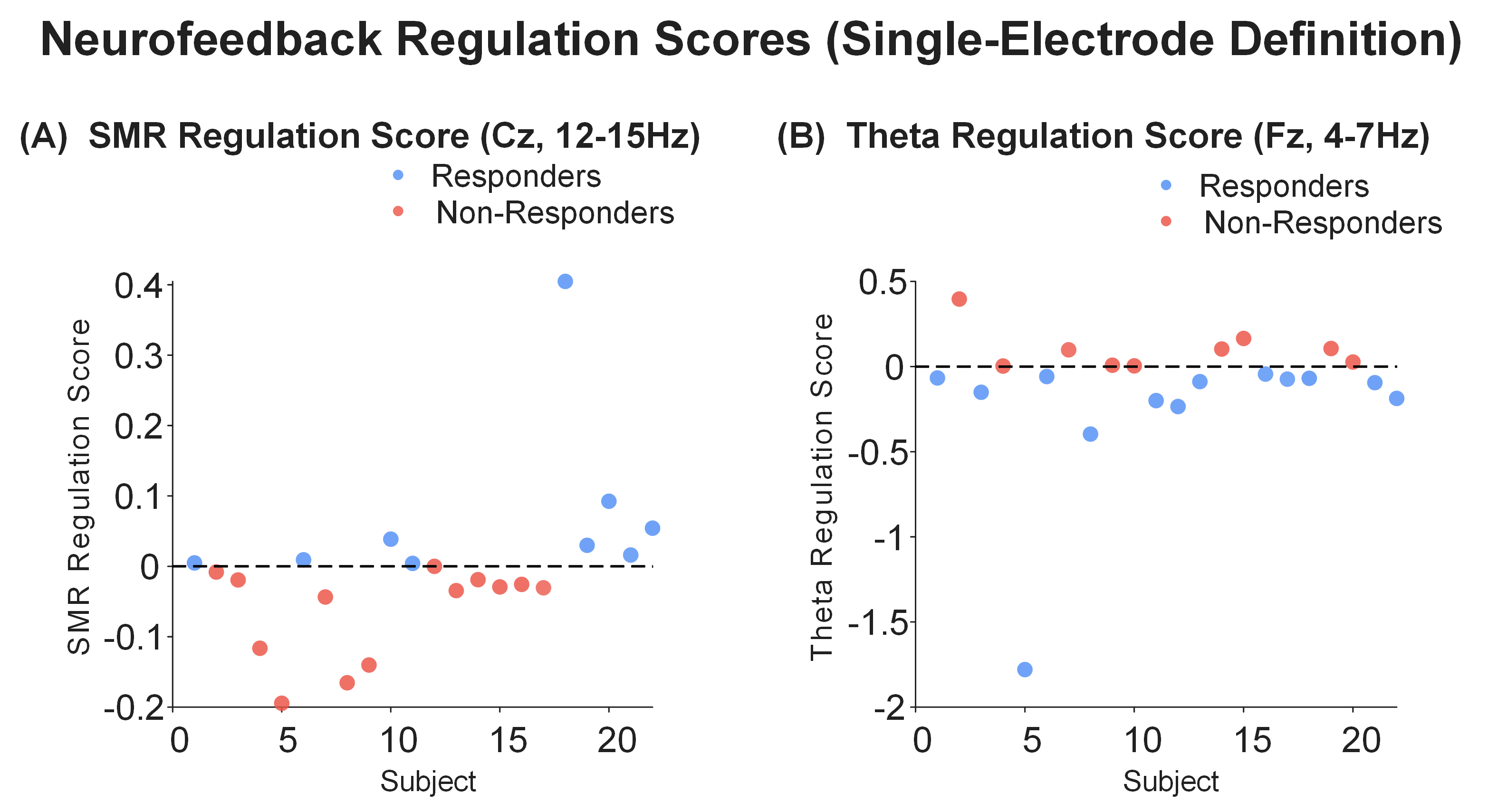

Supplement: Supplementary file 1 [file sensors-26-03187-s001.zip › Supplementary/Figure_S6_RegulationScores.png]

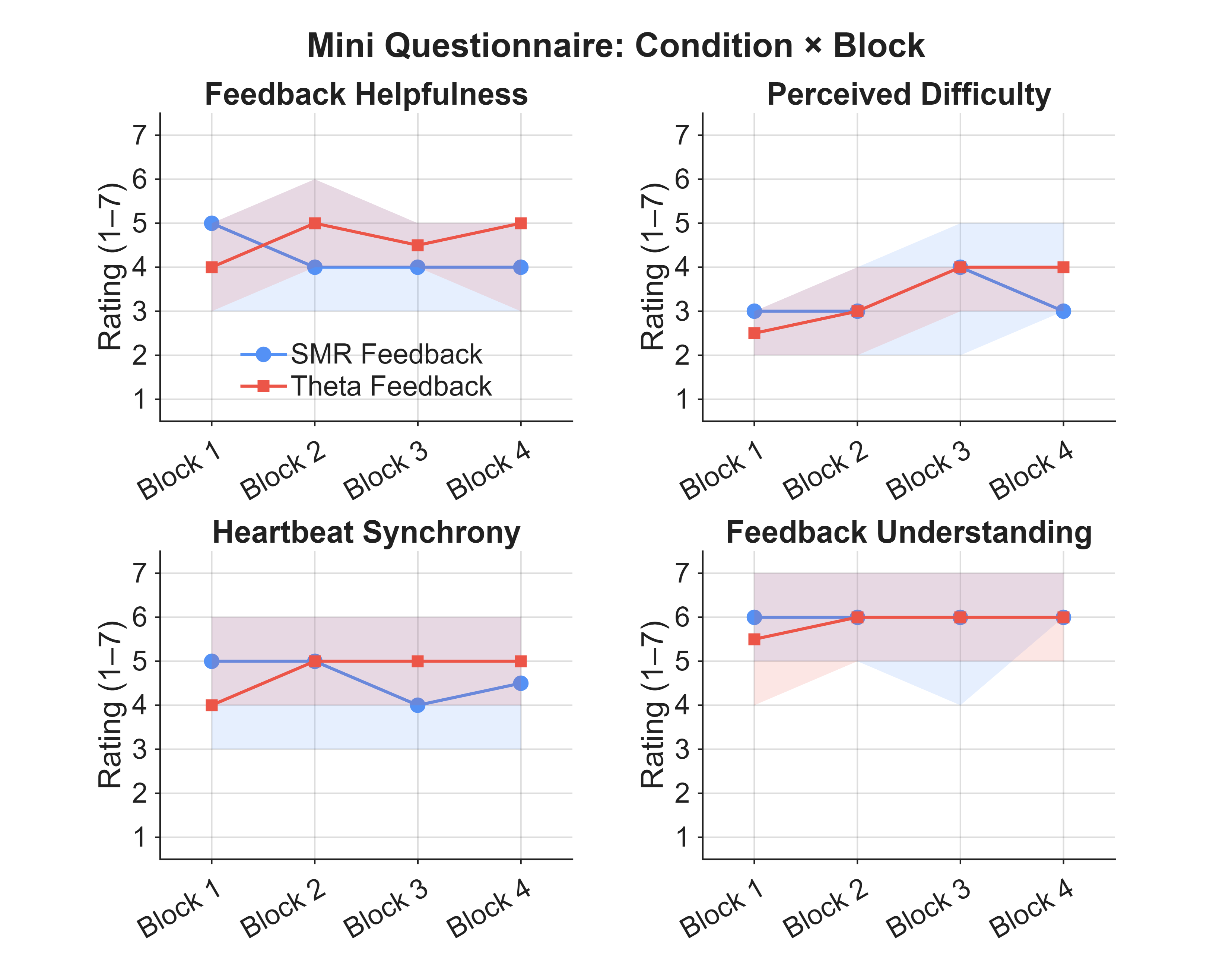

Supplement: Supplementary file 1 [file sensors-26-03187-s001.zip › Supplementary/Figure_S7_MiniQ.png]
